# ﻿On nine ground spiders from Xishuangbanna, China (Araneae, Gnaphosidae), including two new genera and seven new species

**DOI:** 10.3897/zookeys.1174.106340

**Published:** 2023-08-08

**Authors:** YeJie Lin, Shuqiang Li

**Affiliations:** 1 Hebei Key Laboratory of Animal Diversity, College of Life Science, Langfang Normal University, Langfang 065000, China Langfang Normal University Langfang China; 2 Institute of Zoology, Chinese Academy of Sciences, Beijing 100101, China Institute of Zoology, Chinese Academy of sciences Beijing China

**Keywords:** Asia, diagnosis, taxonomy, type, Yunnan

## Abstract

Species of the family Gnaphosidae Banks, 1892 were surveyed in Xishuangbanna Tropical Botanical Garden, and nine species were found including two new genera and seven new species. The new monotypic genera are *Meizhelan***gen. nov.**, with the type species *Meizhelanmuhong***sp. nov.** (♂♀) and *Yuqilin***gen. nov.**, with the type species *Y.lujunyi***sp. nov.** (♂♀). Five additional new species are described: *Allomicythussuochao***sp. nov.** (♂♀); *Hongkongialiutang***sp. nov.** (♂♀); *Sernokorbaruanxiaoer***sp. nov.** (♂♀), *Synaphosusleiheng***sp. nov.** (♂♀) and *Sy.lijun***sp. nov.** (♂♀). The unknown male of *A.kamurai* Ono, 2009 and unknown female of *H.wuae* Song & Zhu, 1998 are described for the first time.

## ﻿Introduction

Gnaphosidae Banks, 1892 or ground spiders, are the fifth largest spider family, with 2443 species in 147 genera worldwide (WSC 2023). Of the 6208 species of spiders described from China, 219 are gnaphosid spiders (pers. obs., cf. Li 2020). Since the publication of a monograph on Gnaphosidae in 2004 (Song et al. 2004), research in China has come to a standstill, with only 28 new species having been published after 2004. Considering the extreme richness of spider biodiversity in China, an enormous number of new species remain undiscovered, especially in the Gnaphosidae.

The Xishuangbanna Tropical Botanical Garden (XTBG, 1125-hectare area) is considered one of the most significant tropical rainforest nature reserves, located on Hulu Island in Menglun Township, Mengla County, at the triple borders of Myanmar, Laos, and Thailand. Research on Gnaphosidae in these three countries is relatively weak, with only 20 species known from Myanmar, two species known from Laos, and nine species known from Thailand, so studies on gnaphosid spiders at the XTBG will help us to understand the biodiversity in neighboring countries. From 2006 to 2023, more than 800 spider species have been reported from XTBG (Li 2021; Yao and Li 2021). From our long-term study, we expect to find more than 1000 spider species from XTBG.

While studying materials of gnaphosid spiders from XTBG, we found 20 species. The goal of this paper is to report nine of them, including two new genera and seven new species.

## ﻿Materials and methods

All specimens were preserved in 80% ethanol. The spermathecae were cleared in trypsin enzyme solution to dissolve non-chitinous tissues. Specimens were examined under a LEICA M205C stereomicroscope. Photomicrographs were taken with an Olympus C7070 zoom digital camera (7.1 megapixels). Photographs were stacked with Helicon Focus® (v. 7.6.1) or Zerene Stacker® (v. 1.04) and processed in Adobe Photoshop CC2022®.

All measurements are in millimetres (mm) and were obtained with an Olympus SZX16 stereomicroscope with a Zongyuan CCD industrial camera. All measurements of body lengths do not include the chelicerae. Eye sizes are measured as the maximum diameter from either the dorsal or the frontal view. Legs were measured laterally. Leg measurements are given as follows: total length (femur, patella+tibia, metatarsus, tarsus). The terminology used in the text and figures follows Marusik and Omelko (2018) and Zhang et al. (2009).

Some of the new genera and species are names from the novel ‘Outlaws of the Marsh’. ‘Outlaws of the Marsh’ (pinyin: Shuǐhǔ Zhuàn), sometimes also translated as ‘Water Margin’ or ‘All Men Are Brothers’, is one of the four most famous works of classical Chinese literature attributed to Shi Nai’an and Luo Guanzhong. The novel details the trials and tribulations of 108 outlaws during the early 12^th^ century.

Types from the current study are deposited in the Institute of Zoology, Chinese Academy of Sciences in Beijing (**IZCAS**).

Abbreviations used in text and figures: **ALE** anterior lateral eye; **AME** anterior median eye; **B** bursa; **C** conductor; **CD** copulatory duct; **CDi** copulatory duct anterior incision; **CO** copulatory opening; **E** embolus; **EA** embolic apophysis; **EF** epigynal fold; **EP** embolus proper; **FD** fertilization duct; **GA** glandular appendage; **H** hood; **MS** median septum; **P** pocket; **PLE** posterior lateral eye; **PME** posterior median eye; **PS** primary spermatheca; **RPO** retro-proximal cymbial outgrowth; **RTA** retrolateral tibial apophysis; **RTH** retrolateral tibial hood; **S** spermatheca; **SC** scape; **SD** sperm duct; **SS** secondary spermathecae; **ST** subtegulum; **TA** tegular apophysis.

## ﻿Taxonomic account

### ﻿Family Gnaphosidae Banks, 1892

#### ﻿Genus Allomicythus

Taxon classificationAnimaliaAraneaeGnaphosidae

Ono, 2009

8D1D1F25-5319-5D5B-95DF-1B0D57EB263D

##### Type species.

*Allomicythuskamurai* Ono, 2009, from Vietnam.

##### Diagnosis.

See Ono (2009).

##### Comments.

This genus belongs to the subfamily Echeminae Simon, 1893, includes two species: *Allomicythuskamurai* and *A.suochao* sp. nov.

##### Distribution.

China and Vietnam.

#### ﻿ Allomicythus kamurai

Taxon classificationAnimaliaAraneaeGnaphosidae

Ono, 2009

A501B67B-A17E-5191-A239-39F70EB059DF

Figs 1A–C , 4A

Allomicythus kamurai Ono, 2009: 6, figs 11–19.

##### Type material.

***Holotype***: ♀ (NSMT-Ar8351), Vietnam: Phu Quoc Island, Duong Dong, 40 m, 19.III.2009, H. Ono leg., not examined.

##### Other material examined.

3♂1♀ (IZCAS-Ar44427–Ar44430), China, Yunnan: Menglun Town: Xishuangbanna Botanical Garden, 21.910767°N, 101.2709°E, ca 572 m, 1–15.V.2007, rubber-tea plantation, fogging. Guo Zheng leg.

##### Diagnosis.

**Figure 1.**
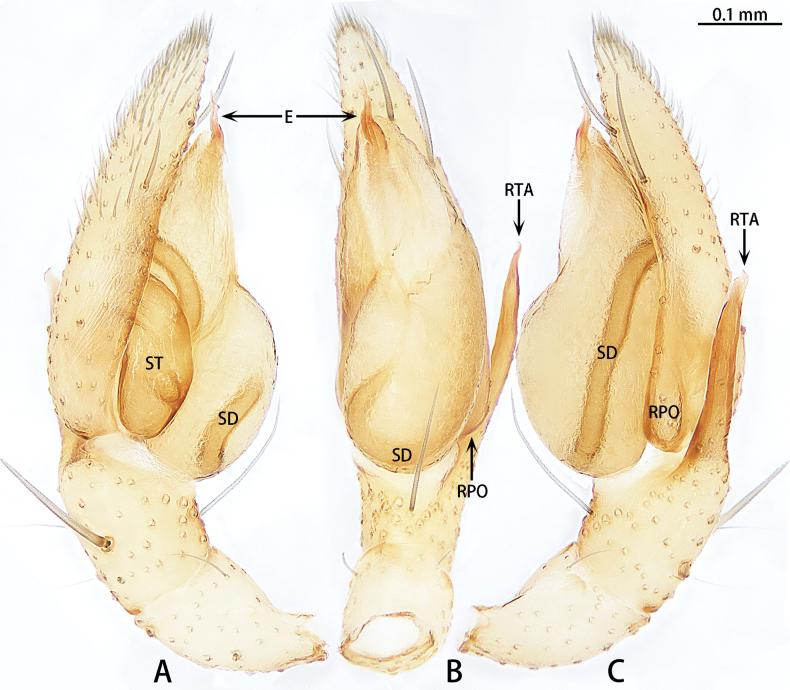
*Allomicythuskamurai*, male **A** prolateral view **B** ventral view **C** retrolateral view. Abbreviations: E = embolus, RPO = retro-proximal cymbial outgrowth, RTA = retrolateral tibial apophysis, SD = sperm duct, ST = subtegulum.

**Figure 2.**
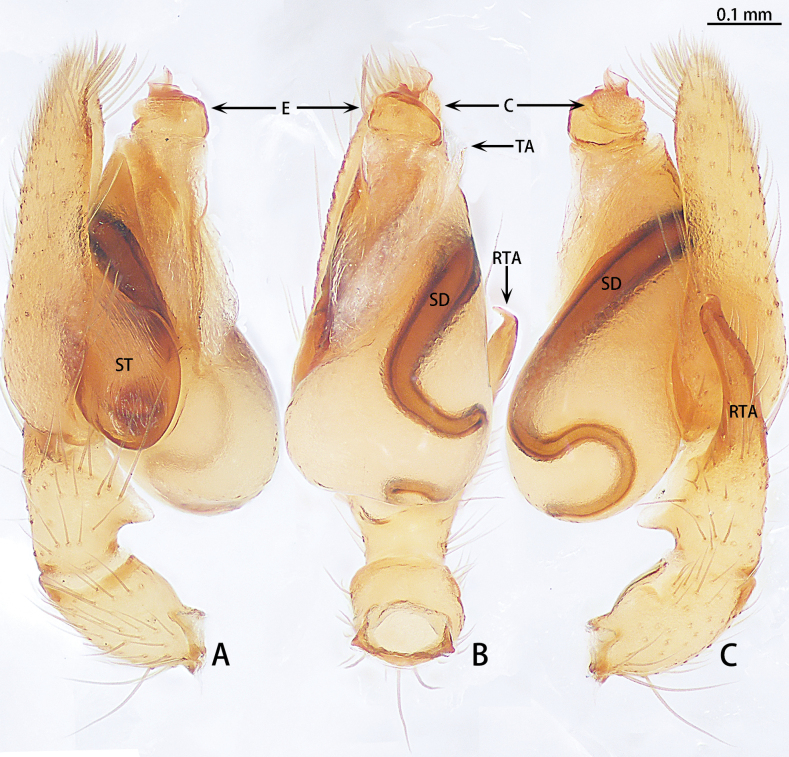
*Allomicythussuochao* sp. nov., holotype male **A** prolateral view **B** ventral view **C** retrolateral view. Abbreviations: C = conductor, E = embolus, RTA = retrolateral tibial apophysis, SD = sperm duct, ST = subtegulum, TA = tegular apophysis.

**Figure 3.**
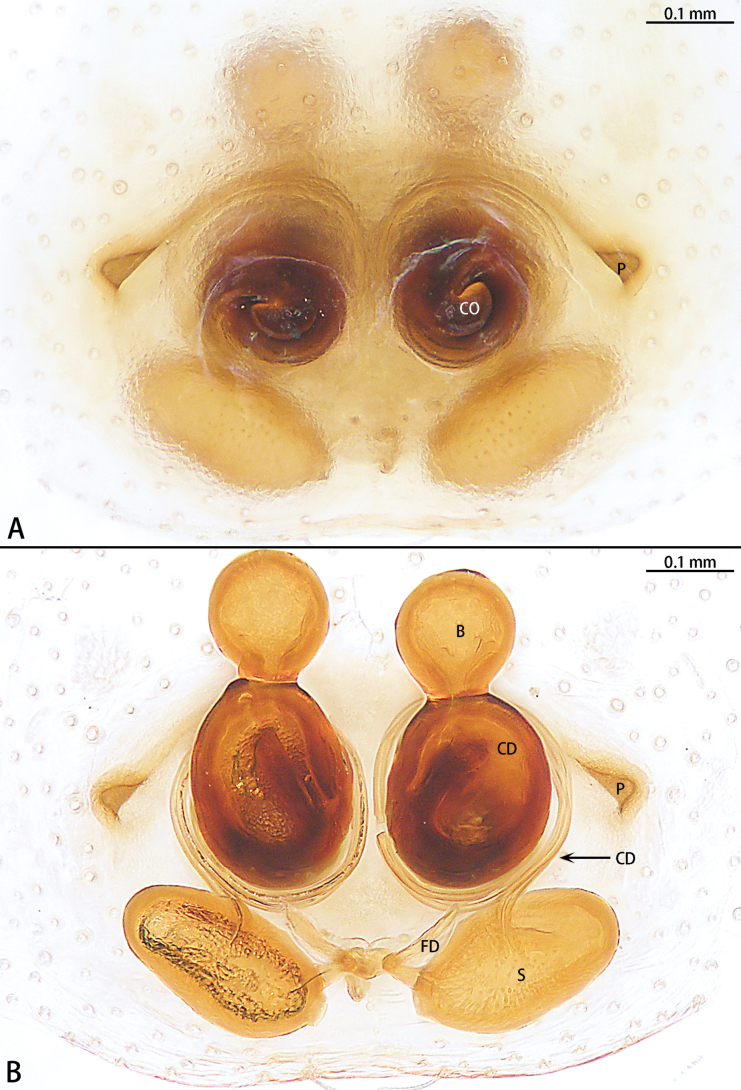
*Allomicythussuochao* sp. nov., paratype female **A** epigyne, ventral view **B** vulva, dorsal view. Abbreviations: B = bursa, CD = copulatory duct, CO = copulatory opening, FD = fertilization duct, P = pocket, S = spermatheca.

The male can be distinguished from *Allomicythussuochao* sp. nov. by the absence of tegular apophysis and conductor (Fig. 1B, C) [vs present (Fig. 2B, C)]. The female can be distinguished from *A.suochao* sp. nov. by the absence of a pair of lateral pockets of epigyne and spermatheca as long as the diameter of the bursa (see Ono 2009: figs 18, 19) [vs lateral pockets present and spermatheca almost twice as long as the diameter of bursa (Fig. 3)].

##### Description.

**Figure 4.**
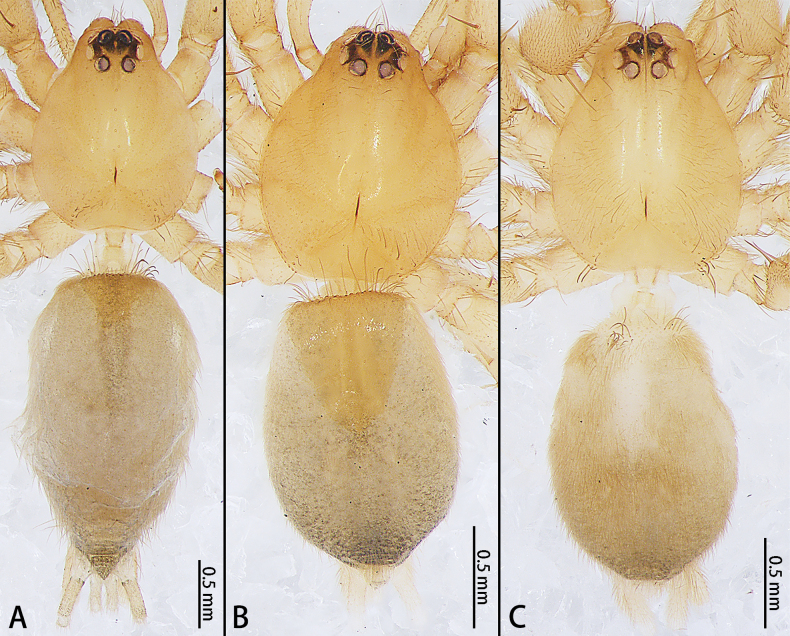
*Allomicythuskamurai* (**A**) and *A.suochao* sp. nov., habitus (**B**, **C**), dorsal view. **A** male **B** male holotype **C** female paratype.

**Male** (IZCAS-Ar44427) (Fig. 4A). Total length 3.40; carapace 1.04 long, 1.22 wide, opisthosoma 1.86 long, 1.03 wide. Eye sizes and interdistances: AME 0.07, ALE 0.07, PME 0.06, PLE 0.06, AME–AME 0.01, AME–ALE 0.01, PME–PME 0.05, PME–PLE 0.02, AME–PME 0.04, ALE–PLE 0.01. Anterior eye row slightly recurved, posterior eye row procurved. Chelicerae with three promarginal teeth. Tarsus and metatarsus in Leg I and II with scopula ventrally. Leg measurements: I 3.15 (0.96, 1.14, 0.60, 0.45), II 3.07 (0.93, 1.10, 0.60, 0.44), III 2.65 (0.72, 0.89, 0.60, 0.44), IV 3.76 (0.96, 1.28, 0.97, 0.55). Opisthosoma oval, with dorsal scutum 1/5 the length of the opisthosoma, venter brown. Spinnerets yellow-brown.

Palp (Fig. 1A–C). Tibia almost as long as patella. Retrolateral tibial apophysis (RTA) long and straight, almost as long as tibia, terminal slightly curved. Cymbium almost 3× longer than wide, with retro-proximal cymbial outgrowth (RPO). Subtegulum (ST) almost oval in prolateral view and unobvious in ventral view. Tegulum almost oblong. Tegular apophysis (TA) and conductor (C) absent. Embolus (E) helically twisted, almost coiled two turns.

**Female.** See Ono (2009).

##### Distribution.

Vietnam, China (Yunnan).

##### Notes.

The male is described here for the first time.

#### ﻿ Allomicythus suochao sp. nov.

Taxon classificationAnimaliaAraneaeGnaphosidae

8CB4C91B-2572-506D-87DB-FEB4FD2784EF

https://zoobank.org/A88D9B9E-6705-4C1F-A070-CC746FDE344C

Figs 2A–C , 3A, B , 4B, C

##### Type material.

***Holotype***: ♂ (IZCAS-Ar44431), China, Yunnan: Menglun Town: Xishuangbanna, Tropical Botanical Garden, 21.9033°N, 101.2820°E, ca 608 m, 16–30.IV.2007, *Paramicheliabaillonii* plantation, pitfall traps, Guo Zheng leg. ***Paratypes***: 2♂3♀ (IZCAS-Ar44432–Ar44436), same data as holotype.

##### Diagnosis.

The male can be distinguished from *Allomicythuskamurai* Ono, 2009 by the presence of tegular apophysis and conductor (Fig. 2B, C) [vs absent (Fig. 1B, C)]. The female can be distinguished from *A.kamurai* by the presence of a pair of lateral pockets of epigyne and spermatheca twice as long as the diameter of the bursa (Fig. 3) [vs lateral pocket absent and spermatheca almost as long as the diameter of bursa (see Ono 2009: figs 18, 19)].

##### Description.

**Male holotype** (Fig. 4B). Total length 2.58; carapace 1.19 long, 0.97 wide, opisthosoma 1.32 long, 0.94 wide. Eye sizes and interdistances: AME 0.06, ALE 0.06, PME 0.06, PLE 0.06, AME–AME 0.02, AME–ALE 0, PME–PME 0.04, PME–PLE 0.02, AME–PME 0.06, ALE–PLE 0.01. Anterior eye row slightly procurved, posterior eye row procurved. Chelicerae with three promarginal and one retromarginal teeth. Tarsus and metatarsus in leg I and II with scopula ventrally. Leg measurements: I 2.54 (0.81, 0.97, 0.39, 0.37), II 2.55 (0.79, 0.93, 0.46, 0.37), III 2.23 (0.63, 0.76, 0.49, 0.35), IV 3.22 (0.90, 1.12, 0.74, 0.46). Opisthosoma oval, with dorsal scutum 1/2 the length of the opisthosoma, venter brown. Spinnerets pale yellow.

Palp (Fig. 2A–C). Tibia almost as long as patella, with a triangular apophysis anteriorly, obtuse, dorsal part lighter in color. Retrolateral tibial apophysis (RTA) long, slightly curved, almost as long as tibia, terminal folded. Cymbium almost 2× longer than wide. Subtegulum (ST) almost oval in prolateral view and unobvious in ventral view. Tegulum teardrop-shaped. Sperm duct (SD) with S-shaped turn in retrolateral view. Tegular apophysis (TA) membranous. Conductor (C) almost spherical. Embolus (E) helically twisted, coiled almost 2.5 turns.

**Female paratype** (IZCAS-Ar44436) (Fig. 4C). Total length 3.04; carapace 1.30 long, 1.06 wide, opisthosoma 1.51 long, 1.02 wide. Eye sizes and interdistances: AME 0.11, ALE 0.10, PME 0.09, PLE 0.10, AME–AME 0.02, AME–ALE 0, PME–PME 0.06, PME–PLE 0.02, AME–PME 0.08, ALE–PLE 0.01. Anterior eye row slightly procurved, posterior eye row procurved Chelicerae with three promarginal teeth. Tarsus and metatarsus in Leg I and II with scopula. Leg measurements: I 2.80 (0.85, 1.07, 0.49, 0.39), II 2.91 (0.94, 1.06, 0.53, 0.38), III 2.51 (0.74, 0.89, 0.47, 0.41), IV 3.36 (0.91, 1.19, 0.76, 0.50). Opisthosoma oval, venter yellow-brown, without scutum. Spinnerets pale yellow.

Epigyne (Fig. 3A, B). Epigynal plate as long as wide, with pair of lateral pockets (P) medially, the lateral pocket almost triangle shaped. Copulatory openings (CO) obvious, strongly sclerotized. Beginning of copulatory duct with bursa (B), strongly sclerotized, oval, then becoming elongated, coiled twice around the copulatory openings and connect the middle of spermatheca. Spermathecae (S) transversally oval (ratio 1:2). Fertilization ducts (FD) directed at 11 o’clock position from spermathecae.

##### Distribution.

Known only from the type locality.

##### Etymology.

The species is named after Suo Chao, one of the 108 outlaws in the classical Chinese novel ‘Outlaws of the Marsh’; noun in apposition.

#### ﻿Genus Hongkongia

Taxon classificationAnimaliaAraneaeGnaphosidae

Song & Zhu, 1998

9BDA4BCD-837C-5327-93B1-B2D60D4CB758

##### Type species.

*Hongkongiawuae* Song & Zhu, 1998, from China.

##### Diagnosis.

See Song and Zhu (1998).

##### Comments.

This genus belongs to the subfamily Echeminae Simon, 1893, includes six species: *Hongkongiacaeca* Deeleman-Reinhold, 2001, *H.incincta* (Simon, 1907), *H.liutang* sp. nov., *H.reptrix* Deeleman-Reinhold, 2001, *H.songi* Zhang, Zhu & Tso, 2009 and *H.wuae* Song & Zhu, 1998.

##### Distribution.

Africa and Asia.

#### ﻿ Hongkongia liutang sp. nov.

Taxon classificationAnimaliaAraneaeGnaphosidae

64AA8A3E-5C82-5016-86EA-7A112A808B0D

https://zoobank.org/6F777E11-4189-480B-87C2-CBAA75DC4D8A

**Figure 5.**
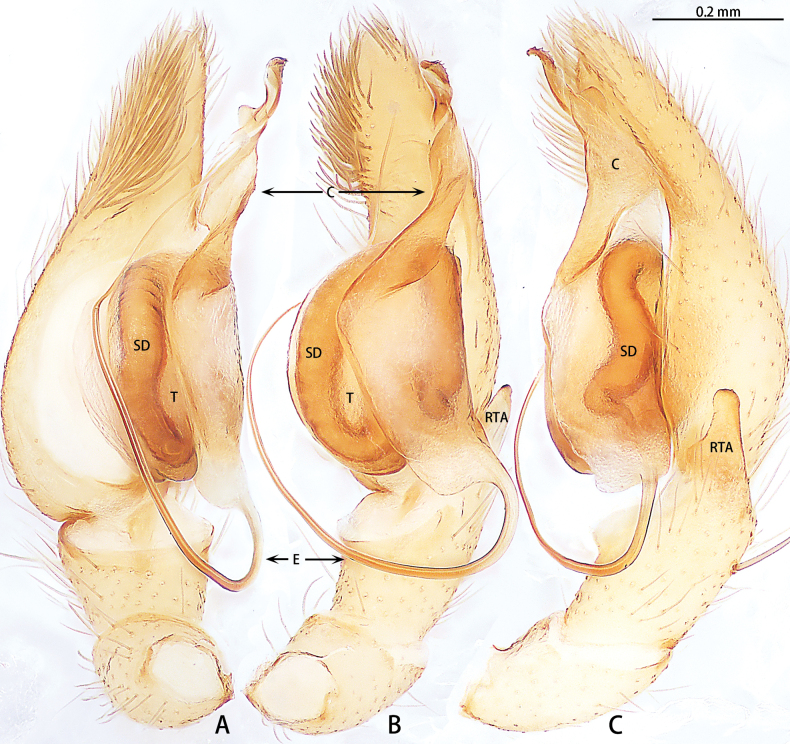
*Hongkongialiutang* sp. nov., holotype male **A** prolateral view **B** ventral view **C** retrolateral view. Abbreviations: C = conductor, E = embolus, RTA = retrolateral tibial apophysis, SD = sperm duct, ST = subtegulum.

**Figure 6.**
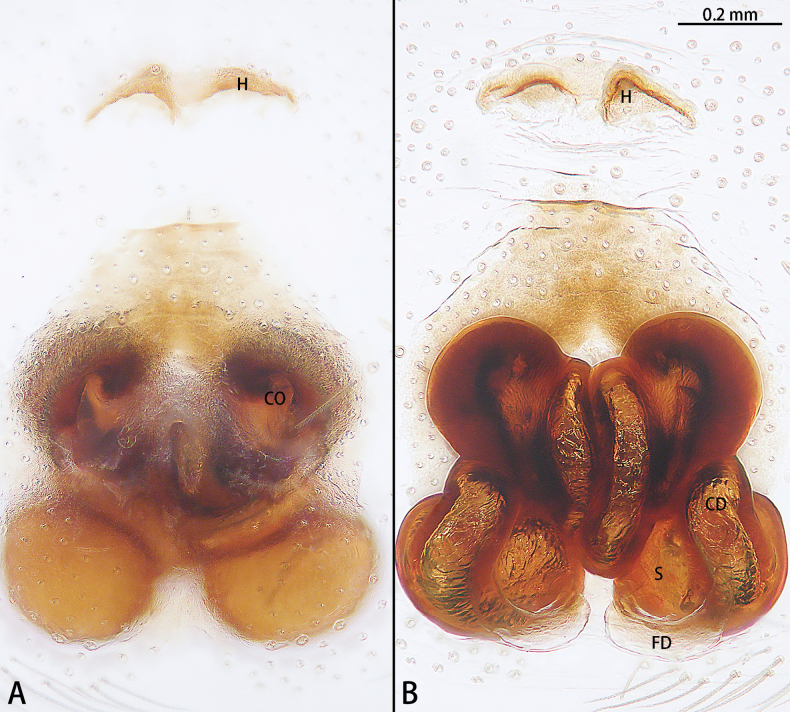
*Hongkongialiutang* sp. nov., paratype female **A** epigyne, ventral view **B** vulva, dorsal view. Abbreviations: CD = copulatory duct, CO = copulatory opening, FD = fertilization duct, H = hood, S = spermatheca.

Figs 5A–C , 6A, B , 8A, B

##### Type material.

***Holotype***: ♂ (IZCAS-Ar44437), China, Yunnan: Menglun Town: Xishuangbanna, Tropical Botanical Garden, 21.9238°N, 101.2740°E, ca 598 m, 1–15.VII.2007, secondary tropical seasonal rain forest, Guo Zheng leg. ***Paratypes***: 5♂6♀ (IZCAS-Ar44438–Ar44448), same data as holotype.

##### Diagnosis.

The male of *Hongkongialiutang* sp. nov. is similar to these of *H.wuae* Song & Zhu, 1998 and *H.reptrix* Deeleman-Reinhold, 2001 by the helical conductor (Fig. 5B). Females of the new species are similar to those of *H.reptrix* by having copulatory ducts subparallel in the middle and close to each other (Fig. 6B). However, the new species can be distinguished from *H.wuae* by having retrolateral tibial apophysis as long as tibia (Fig. 5C) [vs retrolateral tibial apophysis longer than tibia (see Kamura 2019: fig. 2G) and with an outgrowth pointing dorsally (see Song and Zhu 1998: fig. 4E)] and tip of conductor with serrations (Fig. 5B) (vs serrations absent in other congeners). The female of new species can be distinguished from those of *H.reptrix* by the having two hoods (Fig. 6B, C) [vs only one hood (see Zhang et al. 2009: figs 12, 13)].

##### Description.

**Male holotype** (Fig. 8A). Total length 2.84; carapace 1.45 long, 1.06 wide, opisthosoma 1.42 long, 0.90 wide. Eye sizes and interdistances: AME 0.04, ALE 0.05, PME 0.05, PLE 0.04, AME–AME 0.01, AME–ALE 0.01, PME–PME 0.02, PME–PLE 0.01, AME–PME 0.02, ALE–PLE 0.02. Anterior eye row almost straight, posterior eye row recurved. Chelicerae with three promarginal and one retromarginal teeth. Leg measurements: I 4.60 (1.23, 1.80, 0.89, 0.68), II 3.60 (0.98, 1.31, 0.68, 0.63), III 3.10 (0.89, 0.95, 0.73, 0.53), IV 4.15 (1.07, 1.37, 1.00, 0.71). Opisthosoma oval, without scutum, venter dark brown. Spinnerets pale yellow.

Palp (Fig. 2A–C). Femur unmodified. Tibia slightly shorter than patella. Retrolateral tibial apophysis (RTA) 2× shorter than tibia, tip blunt. Cymbium almost 2×longer than wide. Subtegulum absent. Tegulum (T) obvious, almost 1.5× wider than long in ventral view. Conductor (C) and embolus (E) have the same base. Conductor helically twisted, middle with an apophysis, terminal serrated, slightly curved. Embolus directed at 5 o’clock position, whiplike.

**Female paratype** (IZCAS-Ar44444) (Fig. 8B). Total length 4.07; carapace 1.68 long, 1.28 wide, opisthosoma 2.48 long, 1.53 wide. Eye sizes and interdistances: AME 0.13, ALE 0.12, PME 0.12, PLE 0.11, AME–AME 0.02, AME–ALE 0.01, PME–PME 0.04, PME–PLE 0.02, AME–PME 0.06, ALE–PLE 0.02. Chelicerae with three promarginal and one retromarginal teeth. Leg measurements: I 4.27 (1.24, 1.54, 0.83, 0.66), II 3.55 (1.08, 1.21, 0.70, 0.56), III 3.32 (1.00, 1.08, 0.74, 0.50), IV 4.68 (1.23, 1.58, 1.21, 0.66). Opisthosoma oval, without scutum, venter yellow-brown. Spinnerets pale yellow.

Epigyne (Fig. 6A, B). Epigynal plate 1.5× longer than wide, with a pair of hoods (H) anteriorly, hoods almost triangle shaped. Copulatory openings (CO) strongly sclerotized. Copulatory ducts (CD) intertwined. Spermathecae (S) oval. Fertilization ducts (FD) sickle-shaped, directed at 4 o’clock position from spermathecae.

##### Distribution.

Known only from the type locality.

##### Etymology.

The species is named after Liu Tang, one of the 108 outlaws in the classical Chinese novel ‘Outlaws of the Marsh’; noun in apposition.

#### ﻿ Hongkongia wuae

Taxon classificationAnimaliaAraneaeGnaphosidae

Song & Zhu, 1998

556A6D01-3490-5A6A-9720-EAC4375C4C96

Figs 7A, B , 8C

Hongkongia wuae Song & Zhu, 1998: 104, fig. 1A–E (♂); Song et al. 1999: 452, fig. 263P (♂); Deeleman-Reinhold, 2001: 518, figs 886–888 (♂); Song et al. 2004: 157, fig. 92A–E (♂); Zhang et al. 2009: 63, figs 1–7 (♂).

##### Type material.

***Holotype***: ♂, China, Hongkong: New Territories, Duong Dong, 40 m, 19.III.2009, K.-Y. Wu, not examined.

##### Other material examined.

3♂4♀ (IZCAS-Ar44449–Ar44455), China, Yunnan: Menglun Town: Xishuangbanna, Tropical Botanical Garden, 21.9033°N, 101.2820°E, ca 608 m, 16–30.IV.2007, *Paramicheliabaillonii* plantation, pitfall traps, Guo Zheng leg.

##### Diagnosis.

**Figure 7.**
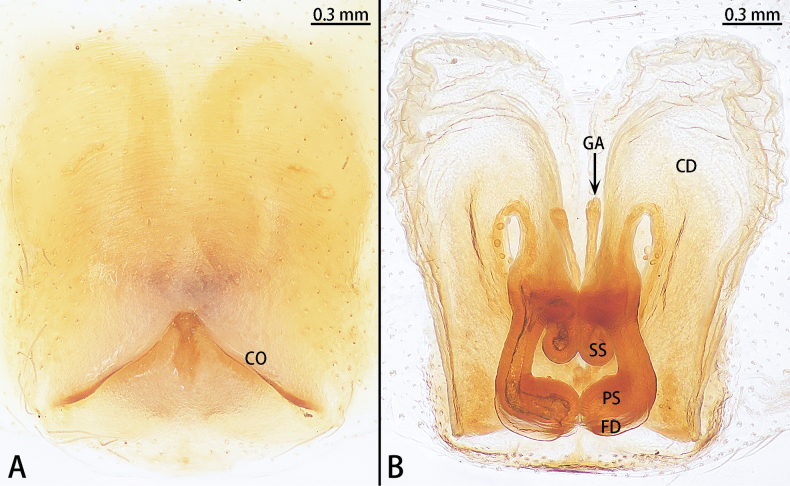
*Hongkongiawuae*, female **A** epigyne, ventral view **B** vulva, dorsal view. Abbreviations: CD = copulatory duct, CO = copulatory opening, FD = fertilization duct, GA = glandular appendage, PS = primary spermatheca, SS = secondary spermathecae.

Females of this species can be easily distinguished from the congeners by the absence of epigynal hood and presence of a long, obvious glandular appendage of spermatheca (Fig. 7A, B). Diagnosis of males see Zhang et al. (2009).

##### Description.

**Figure 8.**
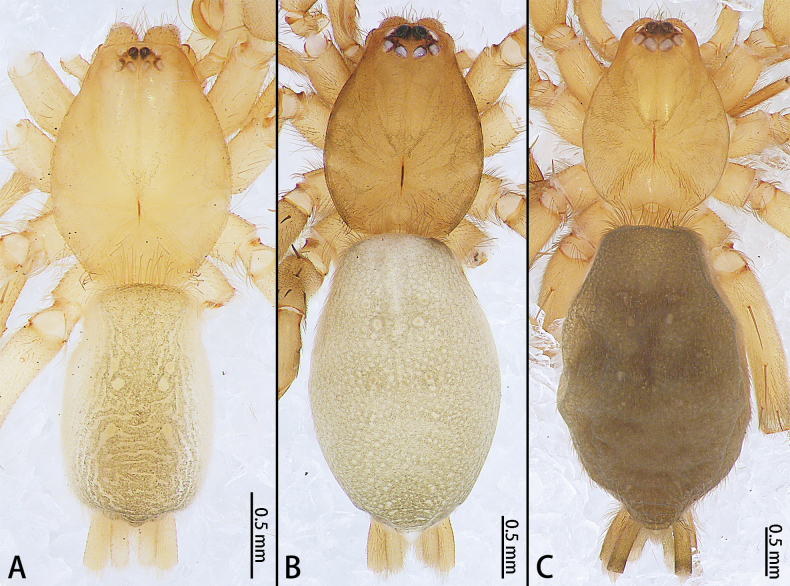
*Hongkongialiutang* sp. nov. (**A, B**) and *H.wuae*, habitus (**C**), dorsal view **A** male holotype **B** female paratype **C** female.

**Female** (IZCAS-Ar44449) (Fig. 8C). Total length 5.25; carapace 1.85 long, 1.52 wide, opisthosoma 3.17 long, 1.95 wide. Eye sizes and interdistances: AME 0.13, ALE 0.14, PME 0.17, PLE 0.16, AME–AME 0.02, AME–ALE 0.01, PME–PME 0, PME–PLE 0.01, AME–PME 0.04, ALE–PLE 0.02. Anterior eye row slightly procurved, posterior eye row recurved. Chelicerae with three promarginal and one retromarginal teeth. Leg measurements: I 5.45 (1.57, 2.02, 1.01, 0.85), II 5.64 (1.59, 2.17, 1.03, 0.85), III 3.98 (1.13, 1.33, 0.91, 0.61), IV 5.87 (1.57, 1.97, 1.37, 0.96). Opisthosoma oval, venter dark brown, without scutum. Spinnerets dark brown.

Epigyne (Fig. 7A, B). Epigynal plate almost 1.5× longer than wide. Copulatory opening (CO) wide, ~ 1/2 epigynal plate width, copulatory openings converging slanting at angle ca 45°, with almost right angle. Copulatory ducts (CD) translucent, n-shaped, median part of copulatory duct with wrinkles. Glandular appendage (GA) straight, directed anteriorly. Secondary spermathecae (SS) oval. Primary spermatheca (PS) elongate, with a 90° bent at two-thirds, terminal parts inflated. Fertilization ducts (FD) sickle-shaped, directed at 2:30 o’clock position from spermathecae.

**Male.** See Song and Zhu (1998).

##### Distribution.

Indonesia (Sulawesi), China (Yunnan, Hongkong).

##### Notes.

The female is described here for the first time.

#### ﻿Genus Meizhelan gen. nov.

Taxon classificationAnimaliaAraneaeGnaphosidae

1B9F3474-31E6-55C0-8B63-5FE08422A8A5

https://zoobank.org/52C560D1-5982-4234-89D8-AD43A69EE6A6

##### Type species.

*Meizhelanmuhong* sp. nov.

##### Diagnosis.

**Figure 9.**
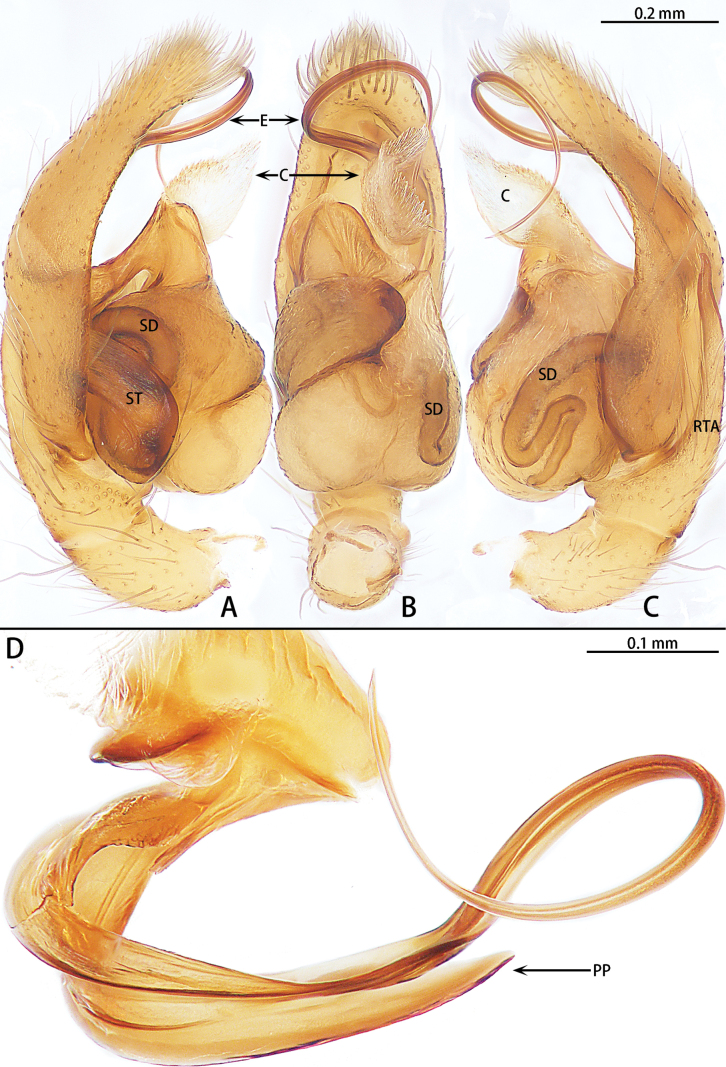
*Meizhelanmuhong* sp. nov., holotype male **A** prolateral view **B** ventral view **C** retrolateral view **D** embolus. Abbreviations: C = conductor, E embolus, PP = paraembolic process, RTA = retrolateral tibial apophysis, SD = sperm duct, ST = subtegulum.

**Figure 10.**
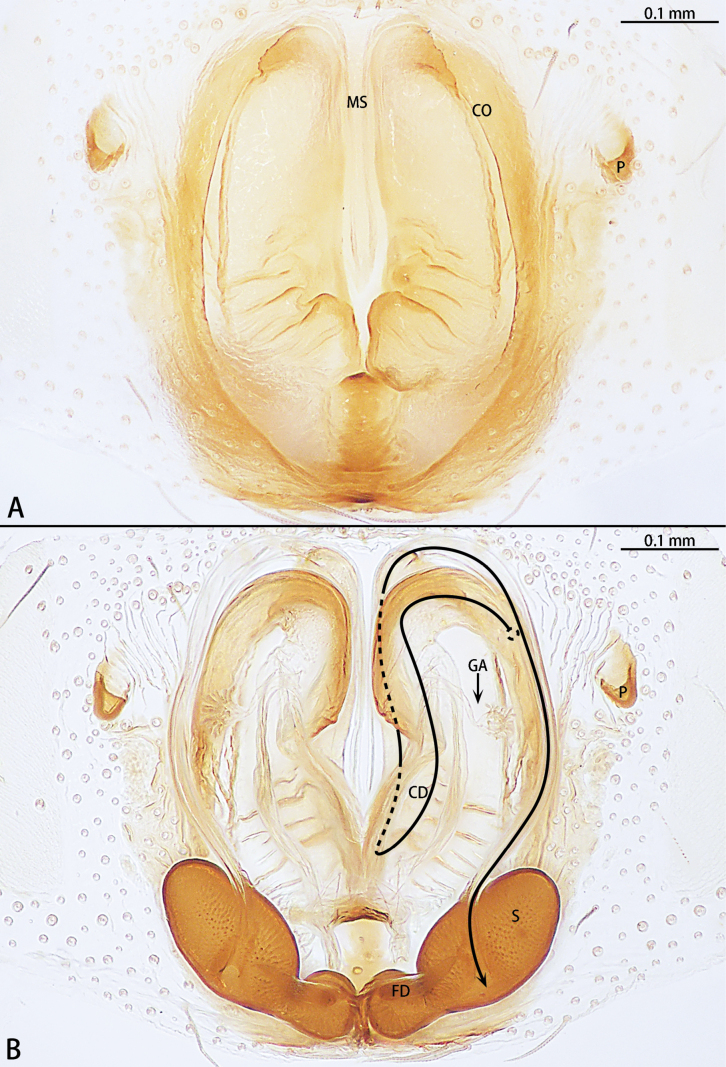
*Meizhelanmuhong* sp. nov., paratype female **A** epigyne, ventral view **B** vulva, dorsal view. Abbreviations: CD = copulatory duct, CO = copulatory opening, FD = fertilization duct, GA = glandular appendage, MS = median septum, P = pocket, S = spermatheca.

*Meizhelan* gen. nov. resemble the *Apodrassodes* Vellard, 1924 (see Platnick and Shadab 1983) by the long, slender embolus (Fig. 9D), membranous conductor (Fig. 9A, B), sperm duct with S-shaped turn (Fig. 9B, C), copulatory duct membranous (Fig. 10B) and spermathecae located posteriorly (Fig. 10B). But it differs in the following: retrolateral tibial apophysis longer than tibia (Fig. 9C) (vs shorter or as long as tibia), absence of tegular apophysis (Fig. 9B) (vs present) and embolus start at the middle of bulb (Fig. 9B) (vs start at posterior of bulb), in the female, spermathecae with two chambers (Fig. 10B) (vs spermathecae oval) and absent of scape and primary spermatheca (Fig. 10A, B) (vs present).

##### Description.

**Figure 11.**
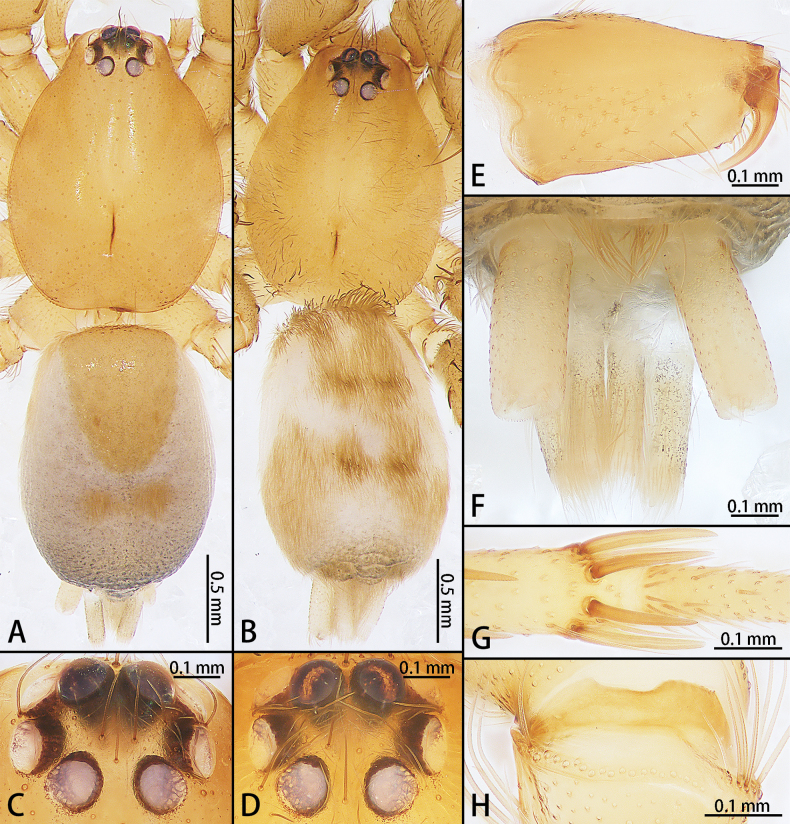
*Meizhelanmuhong* sp. nov., male holotype (**A, C, E–H**) and female paratype (**B, D**) **A, B** habitus, dorsal view **C, D** eye area **E** chelicerae **F** spinnerets **G** metatarsus IV **H** shallow indentation on trochanter.

**Male** (Fig. 11A, C, E–H). Total length 3.19. Carapace yellow brown, covered with brown setae. Fovea longitudinal. Clypeus brown, covered with several plumose setae. Chelicerae yellow-brown, with two promarginal and one retromarginal teeth. Endites pale yellow. Labium pale yellow, covered with brown setae. Sternum colored as endites, covered with brown setae. Legs yellow, without preening comb on metatarsi III and IV, with scopula under claw. Opisthosoma oval, venter pale brown with setae, dorsal scutum almost 1/2 the length of the opisthosoma. Spinnerets pale brown.

Palp as in Fig. 9A–D. Palpal femur almost 4× longer than patella. Tibia 0.6× shorter than patella. Retrolateral tibial apophysis almost 3× longer than tibia. Cymbium almost 3× longer than wide, with fold for retrolateral tibial apophysis. Subtegulum (ST) almost oval in prolateral view. Tegulum almost oval. Sperm duct (SD) with S-shaped turns in ventral and retrolateral view. Tegular apophysis (TA) absent. Conductor (C) membranous, huge, with serrations. Embolus (E) long and slender, with a paraembolic process.

**Female** (Fig. 11B, D). Total length 2.70. Habitus similar to those of male.

Epigynal plate (Fig. 10A, B) with pair of lateral pockets (P), almost triangle shape. Copulatory openings (CO) hidden under anterior edge of epigynal plate, separated by median septum (MS). Copulatory duct (CD) membranous, connect the middle of spermatheca. Spermathecae (S) located posteriorly, with two chambers. Fertilization ducts (FD) start at posterior of spermathecae.

##### Etymology.

The genus is named after Meizhelan, nickname for one of the 108 outlaws in the classical Chinese novel ‘Outlaws of the Marsh’; masculine in gender.

##### Composition.

The new genus currently includes only one species: *Meizhelanmuhong* sp. nov.

##### Distribution.

China (Yunnan).

##### Comments.

This genus belongs to the subfamily Echeminae Simon, 1893.

#### ﻿ Meizhelan muhong sp. nov.

Taxon classificationAnimaliaAraneaeGnaphosidae

4923F841-CD4A-519A-A84D-30BA5A91CC78

https://zoobank.org/2011927D-F61A-45E5-AD29-5ED765024A40

Figs 9A–D , 10A, B , 11A–H

##### Type material.

***Holotype***: ♂ (IZCAS-Ar44456), China, Yunnan: Menglun Town: Xishuangbanna Nature Reserve, 21.9117°N, 101.2816°E, ca 656 m, 13.11.2009, Lùshilin tropical rain forest, fogging, Guo Zheng leg. ***Paratype***: 1♀ (IZCAS-Ar44457), same data as holotype.

##### Diagnosis.

Same as for the genus diagnosis.

##### Description.

**Male holotype** (Fig. 11A, C, E–H). Total length 3.19; carapace 1.61 long, 1.23 wide, opisthosoma 1.47 long, 1.10 wide. Eye sizes and interdistances: AME 0.12, ALE 0.11, PME 0.10, PLE 0.10, AME–AME 0.01, AME–ALE 0, PME–PME 0.07, PME–PLE 0.02, AME–PME 0.10, ALE–PLE 0.02. Anterior eye row procurved, posterior eye row recurved. Chelicerae with two promarginal and one retromarginal teeth. Legs with long brown hairs, with scopula under claw. Leg measurements: I 2.80 (0.91, 1.13, 0.45, 0.31), II 2.80 (0.90, 1.07, 0.46, 0.37), III 2.52 (0.78, 0.83, 0.54, 0.37), IV 3.59 (1.08, 1.24, 0.87, 0.40). Opisthosoma oval, venter brown with long brown setae, dorsal scutum almost 1/2 the length of the opisthosoma. Spinnerets yellow brown.

Palp (Fig. 9A–D). Tibia much shorter than patella. Retrolateral tibial apophysis (RTA) long, tip slightly curved, almost 3× longer than tibia and more than 1/2 of cymbial length. Cymbium almost 3× longer than wide, with fold for retrolateral tibial apophysis. Subtegulum (ST) almost oval in prolateral view. Tegulum almost oval. Sperm duct (SD) with S-shaped turn in retrolateral view. Tegular apophysis (TA) absent. Conductor (C) leaf-shaped, huge, with serrations. Embolus (E) whip-like, with a paraembolic process.

**Female paratype** (IZCAS-Ar44457) (Fig. 11B, D). Total length 2.70; carapace 1.54 long, 1.18 wide, opisthosoma 1.71 long, 1.14 wide. Eye sizes and interdistances: AME 0.12, ALE 0.10, PME 0.11, PLE 0.12, AME–AME 0.03, AME–ALE 0, PME–PME 0.06, PME–PLE 0.02, AME–PME 0.10, ALE–PLE 0.02. Anterior eye row procurved, posterior eye row recurved. Chelicerae as in male. Legs with long brown hairs, with scopula under claw. Leg measurements: I 2.45 (0.82, 0.96, 0.36, 0.31), II 2.69 (0.87, 1.13, 0.36, 0.33), III 2.51 (0.74, 0.85, 0.54, 0.38), IV 3.53 (1.01, 1.23, 0.82, 0.47). Opisthosoma oval, venter yellow-brown with long brown hair. Spinnerets pale yellow.

Epigyne (Fig. 10A, B). Epigynal plate longer than wide, with pair of lateral pockets (P), almost triangle shape. Copulatory openings (CO) indistinct, hidden under edge of epigynal plate, separated by median septum (MS). Copulatory duct (CD) membranous, connected each other with a sclerotized lamella, connect the middle of spermatheca. Spermathecae (S) with two chambers, one large, oval, and other small, globular, the length of the larger one is 3× the diameter of the smaller one. Fertilization ducts (FD) directed at 2.30 o’clock position from spermathecae.

##### Distribution.

Known only from the type locality.

##### Etymology.

The species is named after Mu Hong, one of the 108 outlaws in the classical Chinese literature ‘Outlaws of the Marsh’; noun in apposition.

#### ﻿Genus Sernokorba

Taxon classificationAnimaliaAraneaeGnaphosidae

Kamura, 1992

FE9DA9D8-AE70-5E46-99B3-6DBAFBC84C86

##### Type species.

*Prosthesimapallidipatellis* Bösenberg & Strand, 1906, from Japan.

##### Diagnosis.

See Gallé-Szpisjak et al. (2023).

##### Comments.

This genus belongs to the subfamily Herpyllinae Platnick, 1990, includes five species: *Sernokorbabetyar* Gallé-Szpisjak, Gallé & Szűts, 2023, *S.fanjing* Song, Zhu & Zhang, 2004, *S.pallidipatellis* (Bösenberg & Strand, 1906), *S.ruanxiaoer* sp. nov. and *S.tescorum* (Simon, 1914).

##### Distribution.

Europe and Asia.

#### ﻿ Sernokorba ruanxiaoer sp. nov.

Taxon classificationAnimaliaAraneaeGnaphosidae

53584A4E-248E-5F26-B88A-9EEEE3206CD7

https://zoobank.org/10C0B496-50A8-47DA-90D8-89A646D5B545

**Figure 12.**
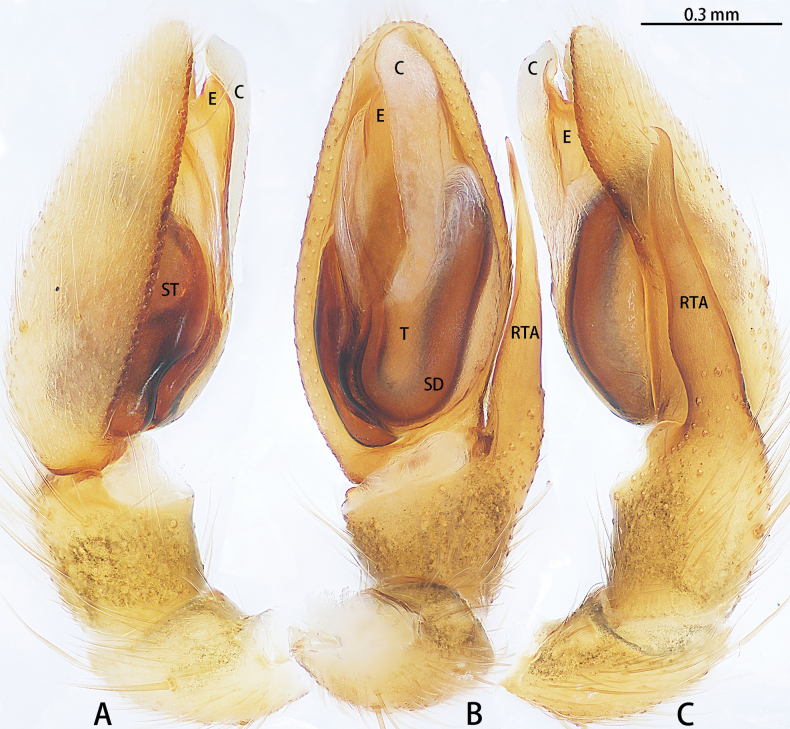
*Sernokorbaruanxiaoer* sp. nov., holotype male **A** prolateral view **B** ventral view **C** retrolateral view. Abbreviations: C = conductor, E = embolus, RTA = retrolateral tibial apophysis, SD = sperm duct, ST = subtegulum, T = tegulum.

**Figure 13.**
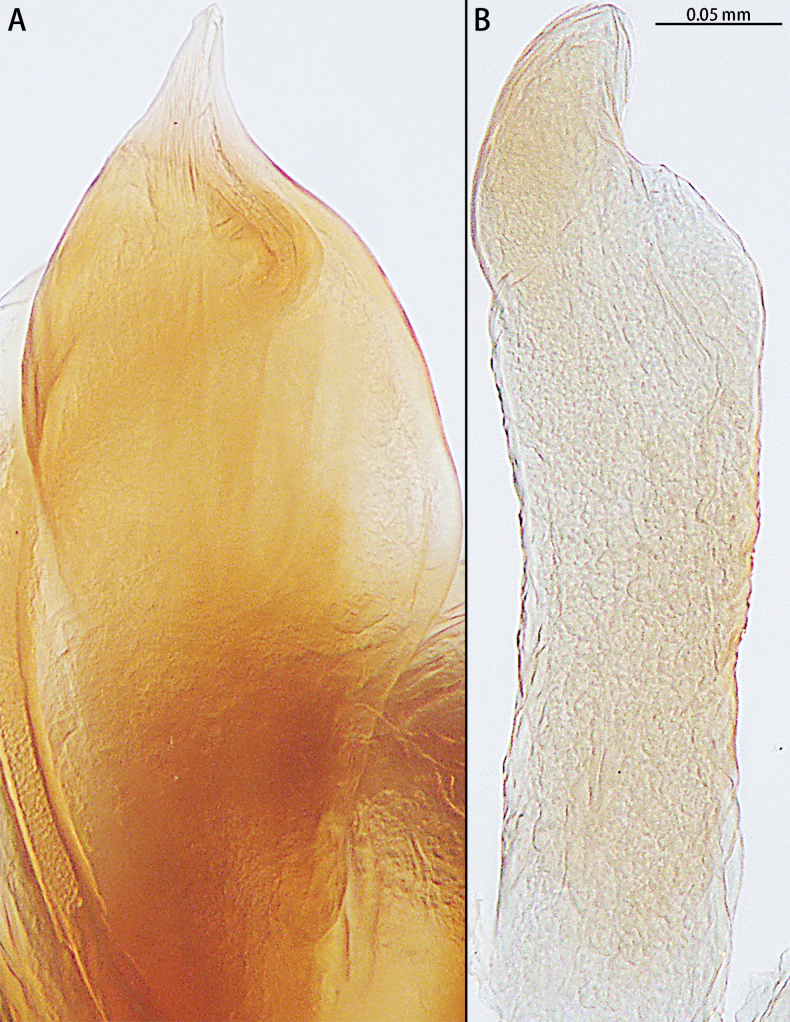
*Sernokorbaruanxiaoer* sp. nov., paratype male **A** embolus, ventral view **B** conductor, ventral view.

**Figure 14.**
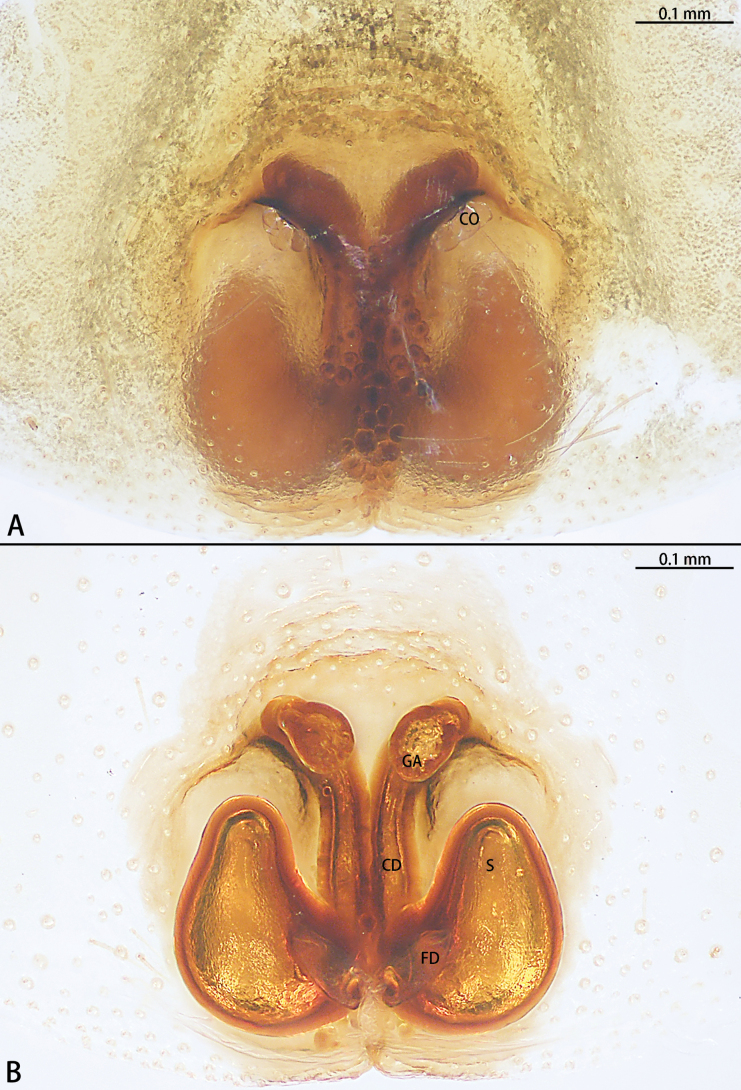
*Sernokorbaruanxiaoer* sp. nov., paratype female **A** epigyne, ventral view **B** vulva, dorsal view. Abbreviations: CD = copulatory duct, CO = copulatory opening, FD = fertilization duct, GA = glandular appendage, S = spermatheca.

**Figure 15.**
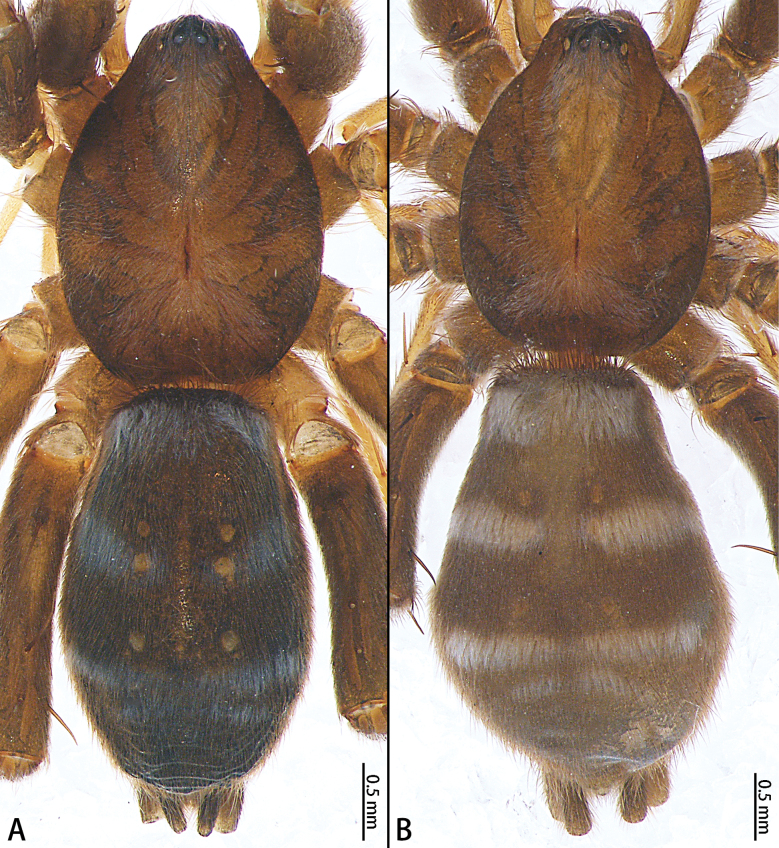
*Sernokorbaruanxiaoer* sp. nov., habitus, dorsal view **A** male holotype **B** female paratype.

Figs 12A–C , 13A, B , 14A, B , 15A, B

##### Type material.

***Holotype***: ♂ (IZCAS-Ar44498), China, Yunnan: Menglun Town: Xishuangbanna Botanical Garden, 21.910767°N, 101.2709°E, ca 572 m, 15–31.II.2007, rubber-tea plantation, fogging. Guo Zheng leg. ***Paratypes***: 2♂1♀ (IZCAS-Ar44499–Ar44501), same data as holotype.

##### Diagnosis.

The male of *Sernokorbaruanxiaoer* sp. nov. is similar to these of *S.fanjing* Song, Zhu & Zhang, 2004 and *S.pallidipatellis* (Bösenberg & Strand, 1906) by the conductor without serration (Figs 12B, 13B). Females of the new species are similar to those of *S.pallidipatellis* by unobvious copulatory openings (Fig. 14A). However, the new species can be distinguished from *S.fanjing* by the tip of the conductor blunt (Figs 12B, 13B) [vs sharped (see Wang et al. 2023: fig. 1B)] and can be distinguished from *S.pallidipatellis* by the tip of the embolus straight (Fig. 13A) [vs curved (see Gallé-Szpisjak et al. 2023: figs 23, 33)]. The female can be distinguished from those of *S.pallidipatellis* by the ratio of the length of the spermathecae to the diameter of the copulatory duct is almost 1:1 and the diameter of the glandular appendage is longer than the diameter of the copulatory duct (Fig. 14B) [vs 2:3 and shorter than the diameter of the copulatory duct (see Kamura 1992: fig. 7)].

##### Description.

**Male holotype** (Fig. 15A). Total length 5.02; carapace 2.41 long, 1.72 wide, opisthosoma 2.48 long, 1.64 wide. Eye sizes and interdistances: AME 0.08, ALE 0.10, PME 0.09, PLE 0.09, AME–AME 0.05, AME–ALE 0.01, PME–PME 0.07, PME–PLE 0.06, AME–PME 0.09, ALE–PLE 0.09. Anterior eye row and posterior eye row recurved. Chelicerae with eight promarginal and one retromarginal teeth. Leg measurements: I 5.56 (1.63, 1.85, 1.14, 0.94), II 5.45 (1.49, 1.79, 1.21, 0.96), III 5.65 (1.58, 1.75, 1.37, 0.95), IV 8.29 (2.09, 2.32, 2.27, 1.61). Opisthosoma elongate-oval, venter dark brown, with four transverse white bands of setae, dorsal scutum almost 1/2 the length of the opisthosoma. Spinnerets dark brown.

Palp (Figs 12A–C, 13A, B). Femur unmodified. Tibia slightly longer than patella. Retrolateral tibial apophysis 1.5× longer than tibia, bent in its middle and with an apical hook. Cymbium almost two× longer than wide. Subtegulum (ST) obvious in prolateral and ventral view. Tegulum obvious, almost 2.5× wider than long in ventral view, with long and U-shaped sperm duct (SD). Conductor (C) membranous, 1/2 the bulb length, encircled embolus distally. Embolus (E) short, originated from the apical portion of bulb, straight.

**Female paratype** (IZCAS-Ar44501) (Fig. 15B). Total length 5.68; carapace 2.53 long, 1.86 wide, opisthosoma 3.13 long, 2.14 wide. Eye sizes and interdistances: AME 0.07, ALE 0.11, PME 0.09, PLE 0.11, AME–AME 0.05, AME–ALE 0.01, PME–PME 0.07, PME–PLE 0.06, AME–PME 0.11, ALE–PLE 0.08. Chelicerae with eight promarginal and one retromarginal teeth. Leg measurements: I 5.46 (1.67, 1.93, 0.97, 0.89), II 5.65 (1.72, 1.84, 1.13, 0.96), III 5.79 (1.54, 1.83, 1.27, 1.15), IV 7.93 (2.06, 2.39, 2.27, 1.21). Opisthosoma as in male but without dorsal scutum. Spinnerets dark brown.

Epigyne (Fig. 14A, B). Epigynal plate almost as long as wide. Copulatory openings (CO) unobvious, the edge of the copulatory openings strongly sclerotized. Copulatory ducts (CD) slightly curved, with glandular appendage (GA) anteriorly. Spermathecae (S) pear-shaped. Fertilization ducts (FD) sickle-shaped, directed at 2 o’clock position from spermathecae.

##### Distribution.

Known only from the type locality.

##### Etymology.

The species is named after Ruan Xiaoer, one of the 108 outlaws in the classical Chinese novel ‘Outlaws of the Marsh’; noun in apposition.

#### ﻿Genus Synaphosus

Taxon classificationAnimaliaAraneaeGnaphosidae

Platnick & Shadab, 1980

7B14F604-A62E-5D2D-9273-5CBEE70BC865

##### Type species.

*Nodocionsyntheticus* Chamberlin, 1924, from USA.

##### Diagnosis.

See Marusik and Omelko (2018).

##### Comments.

This genus remains unassigned to any subfamily or tribe of Gnaphosidae, belonging only to the informal *Echemus* group of genera, includes 37 species, see World Spider Catalog, 2023.

##### Species groups.

Five species groups: the *syntheticus* group, the *gracillimus* group, the *kakamega* group and the *femininis* group. Here, we report a new group: the *dubius*-group with two species: *Synaphosusdubius* Marusik & Omelko, 2018 and *S.lijun* sp. nov. This group can be distinguished by the male embolus originating at 9 o’clock position and females have long, stick-like and inflexible scape and spermathecae with 6–10 turns.

##### Distribution.

Africa, Asia, Europe, and North America.

### ﻿The *syntheticus* group

#### ﻿ Synaphosus leiheng sp. nov.

Taxon classificationAnimaliaAraneaeGnaphosidae

3F1ABF8B-5862-5EB2-ABD1-4380575CAC34

https://zoobank.org/DE6388EC-FDAF-4439-A120-50764E0A88A3

**Figure 16.**
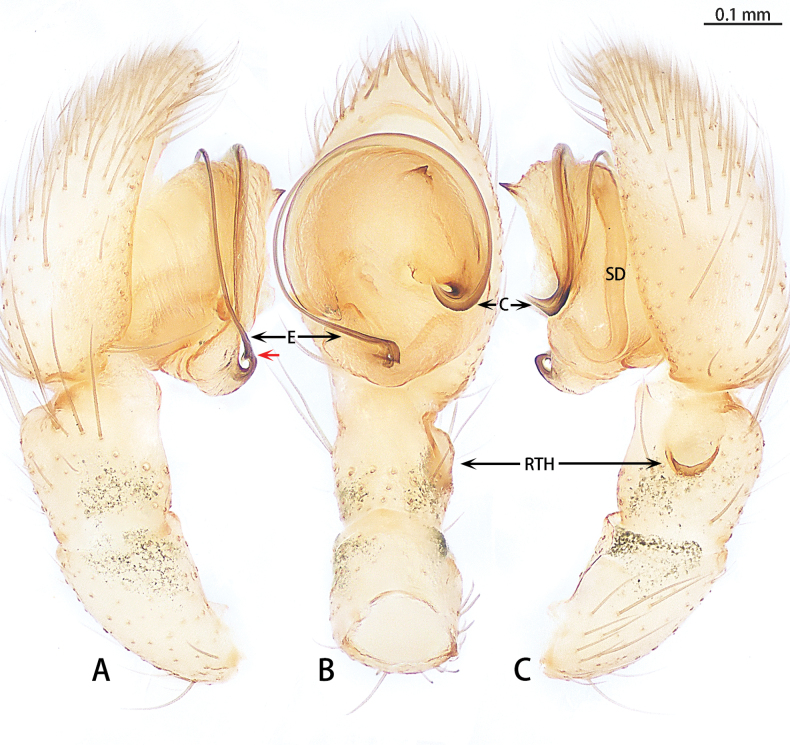
*Synaphosusleiheng* sp. nov., holotype male **A** prolateral view **B** ventral view **C** retrolateral view. Abbreviations: C = conductor, E = embolus, RTH = retrolateral tibial hood, SD = sperm duct. Red arrow shows the apophysis at the base of embolus.

**Figure 17.**
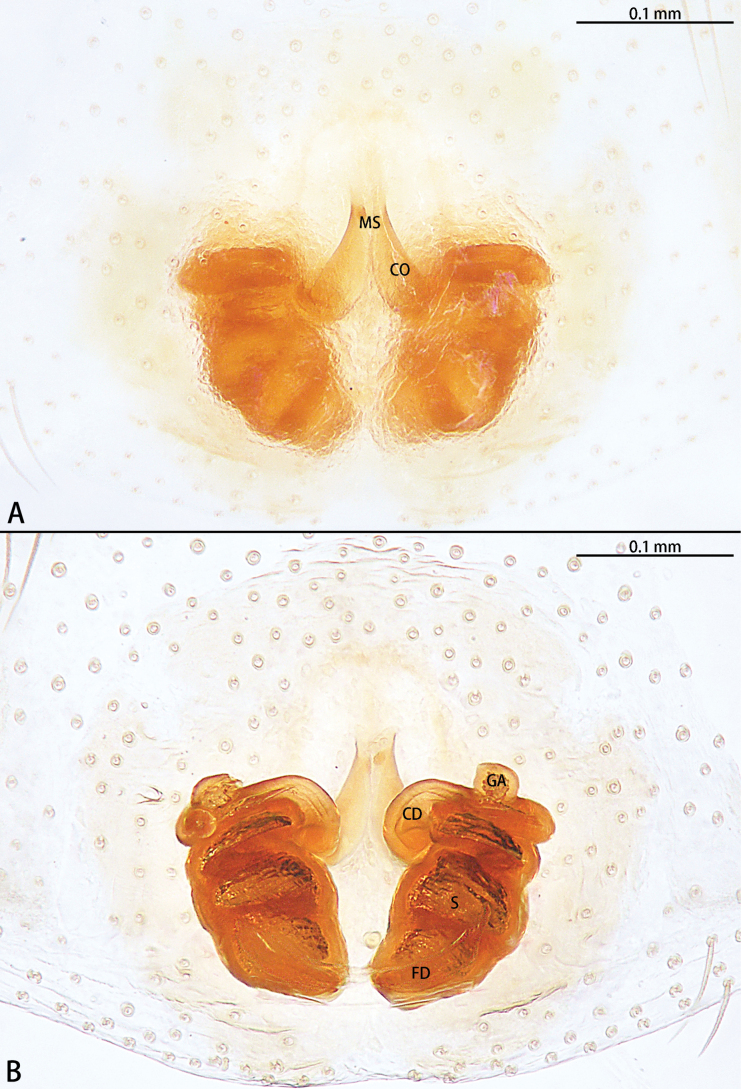
*Synaphosusleiheng* sp. nov., paratype female **A** epigyne, ventral view **B** vulva, dorsal view. Abbreviations: CD = copulatory duct, CO = copulatory opening, FD = fertilization duct, GA = glandular appendage, MS = median septum, S spermatheca.

Figs 16A–C , 17A, B , 20A, B

##### Type material.

***Holotype***: ♂ (IZCAS-Ar44458), China, Yunnan: Menglun Town: Xishuangbanna Nature Reserve, 21.9611°N, 101.1982°E, ca 790 m, 16–24.2006, Guo Zheng leg. ***Paratypes***: 1♀ (IZCAS-Ar44459), same data as holotype; 11♀ (IZCAS-Ar44460–Ar44470), China, Yunnan: Menglun Town: Xishuangbanna Botanical Garden, 21.8970°N, 101.2845°E, ca 613 m, 1–15.II.2007, Guo Zheng leg.

##### Diagnosis.

The new species similar to *S.evertsi* Ovtsharenko, Levy & Platnick, 1994 by the male with a retrolateral tibial hood (Fig. 16B) and lake of retrolateral tibial apophysis (Fig. 16B) and females by the deep, separated, anterior copulatory opening (Fig. 17A). However, the new species can be distinguished by the hook-shaped conductor (Fig. 16B) [vs conductor long and slender (see Marusik and Omelko 2018: fig. 42)], an apophysis at the base of embolus (Fig. 16A) (vs absent) and only one apophysis on conductor (Fig. 16B) [vs two apophyses (see Marusik and Omelko 2018: fig. 42)]. The female can be distinguished by the absence of anterior pockets (Fig. 17A) [vs present (see Ovtsharenko et al. 1994: fig. 51)] and the copulatory ducts connected directly to the anterior part of spermatheca (Fig. 17B) [vs copulatory ducts wrap around spermatheca and then connect to the middle of spermatheca (see Ovtsharenko et al. 1994: fig. 52)].

##### Description.

**Male holotype** (Fig. 20A). Total length 3.13; carapace 1.38 long, 1.05 wide, opisthosoma 1.67 long, 0.92 wide. Eye sizes and interdistances: AME 0.04, ALE 0.04, PME 0.03, PLE 0.04, AME–AME 0.02, AME–ALE 0, PME–PME 0.01, PME–PLE 0.01, AME–PME 0.03, ALE–PLE 0.02. Anterior eye row slightly recurved, posterior eye row straight. Chelicerae with three promarginal and three retromarginal teeth. Legs with long brown hair. Leg measurements: I 4.51 (1.20, 1.83, 0.83, 0.65), II 3.20 (0.90, 1.19, 0.62, 0.49), III 3.53 (1.05, 1.19, 0.62, 0.49), IV 3.21 (0.71, 0.83, 1.11, 0.56). Opisthosoma oval, venter brown with long hair, dorsal scutum absent. Spinnerets pale yellow.

Palp (Fig. 16A–C). Femur and patellar without apophysis. Tibia almost as long as patella. Retrolateral tibial hood (RTH) almost 2× wider than long, terminal part slightly curved. Cymbium almost 1.5× longer than wide. Subtegulum (ST) indistinct, hidden between cymbium and tegulum. Tegulum round. Conductor (C) tip strongly curved, without serration, basal with an apophysis, triangle shaped. Embolus (E) whip-like, basal part with an apophysis, originates at 6 o’clock position.

**Female paratype** (IZCAS-Ar44460) (Fig. 20B). Total length 2.75; carapace 1.26 long, 0.88 wide, opisthosoma 1.54 long, 0.97 wide. Eye sizes and interdistances: AME 0.04, ALE 0.04, PME 0.05, PLE 0.04, AME–AME 0.01, AME–ALE 0.01, PME–PME 0.02, PME–PLE 0.01, AME–PME 0.03, ALE–PLE 0.01. Anterior eye row almost straight, posterior eye row recurved. Chelicerae as in male. Leg measurements: I 2.71 (0.83, 1.02, 0.46, 0.40), II 2.33 (0.71, 0.82, 0.44, 0.36), III 2.03 (0.65, 0.61, 0.42, 0.35), IV 3.07 (0.85, 1.11, 0.69, 0.42). Opisthosoma oval, venter yellow-brown with long brown hair. Spinnerets pale yellow.

Epigyne (Fig. 17A, B). Epigynal plate almost as long as wide. Copulatory openings (CO) located anteriorly, separated by median septum (MS). Copulatory duct (CD) short, connected to anterior part of spermatheca. Glandular appendage (GA) distinct, anteriorly. Spermathecae (S) kidney-shaped, almost 2× wider than long. Fertilization ducts (FD) directed at 2 o’clock position from spermathecae.

##### Distribution.

Known only from the type locality.

##### Etymology.

The species is named after Lei Heng, one of the 108 outlaws in the classical Chinese novel ‘Outlaws of the Marsh’; noun in apposition.

### ﻿The *dubius* group

#### ﻿ Synaphosus lijun sp. nov.

Taxon classificationAnimaliaAraneaeGnaphosidae

3069605B-50FC-5F51-AD13-A434421E17DD

https://zoobank.org/5ADB98E7-7A72-49D1-ABC8-A0C4D446C570

**Figure 18.**
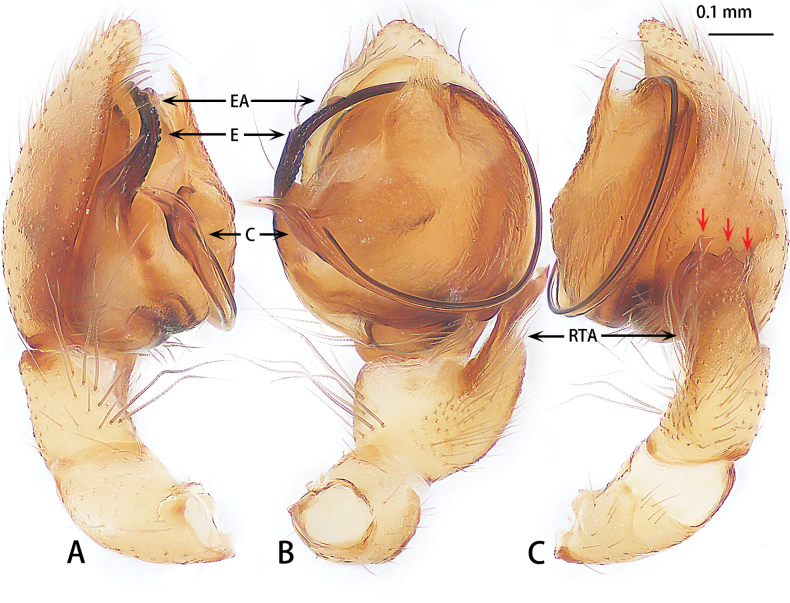
*Synaphosuslijun* sp. nov., holotype male **A** prolateral view **B** ventral view **C** retrolateral view. Abbreviations: C = conductor, E = embolus, EA = embolic apophysis, RTA = retrolateral tibial apophysis. Red arrows show the outgrowths on the retrolateral tibial apophysis.

**Figure 19.**
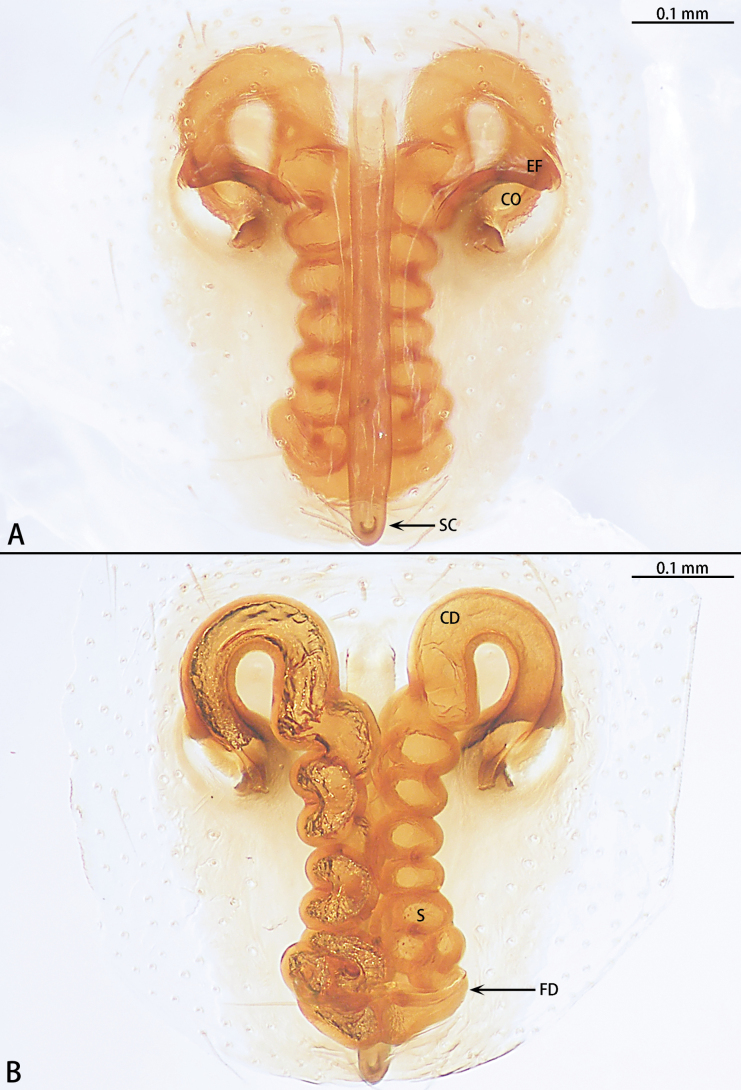
*Synaphosuslijun* sp. nov., paratype female **A** epigyne, ventral view **B** vulva, dorsal view. Abbreviations: CD = copulatory duct, CO = copulatory opening, EF = epigynal fold, FD = fertilization duct, SC = scape, S = spermatheca.

**Figure 20.**
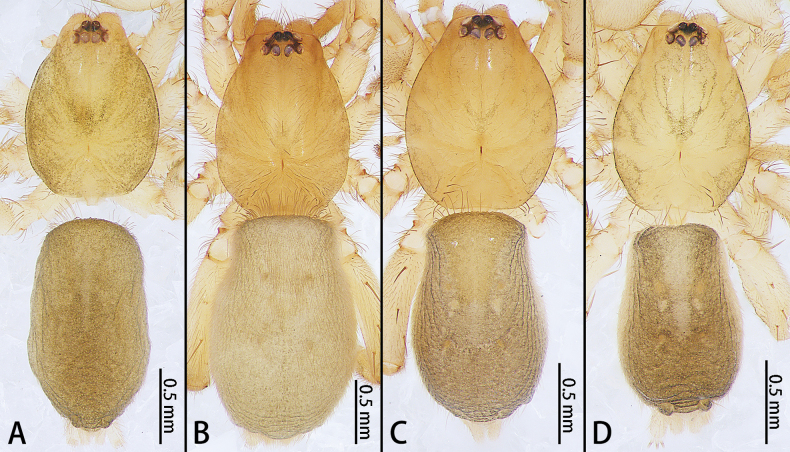
*Synaphosusleiheng* sp. nov. (**A, B**) and *S.lijun* sp. nov., habitus (**C, D**), dorsal view **A** male holotype **B** female paratype **C** male holotype **D** female paratype.

Figs 18A–C , 19A, B , 20C, D

##### Type material.

***Holotype***: ♂ (IZCAS-Ar44471), China, Yunnan: Mengla County: Xishuangbanna Nature Reserve, Xiaolongha biodiversity preservation corridor, 21.9129°N, 101.2674°E, ca 556 m, 5–12.X.2006, *Paramicheliabaillonii* plantation, Guo Zheng leg. ***Paratypes***: 7♂8♀ (IZCAS-Ar44472–Ar44486), same data as holotype.

##### Diagnosis.

Male of the new species can be distinguished from all other congeners by the embolus originating at 9 o’clock position (Fig. 18B) (vs at 5–6 o’clock position). Females resemble those of *S.dubius* Marusik & Omelko, 2018 by the long, stick-like and inflexible scape (Fig. 19B), but can be distinguished from *S.dubius* by the presence of epigynal fold (Fig. 19A) [vs absent in *S.dubius* (see Marusik and Omelko 2018: fig. 46)], copulatory ducts separated (Fig. 19B) [vs copulatory ducts touching in *S.dubius* (see Marusik and Omelko 2018: fig. 48)] and spermathecae with ten turns (Fig. 19B) [vs six turns in *S.dubius* (see Marusik and Omelko 2018: fig. 48)].

##### Description.

**Male holotype** (Fig. 20C). Total length 2.56; carapace 1.19 long, 0.94 wide, opisthosoma 1.36 long, 0.84 wide. Eye sizes and interdistances: AME 0.04, ALE 0.05, PME 0.05, PLE 0.04, AME–AME 0.01, AME–ALE 0, PME–PME 0.01, PME–PLE 0.04, AME–PME 0.02, ALE–PLE 0.01. Anterior eye row almost straight, posterior eye row recurved. Chelicerae with three promarginal and one retromarginal teeth. Leg measurements: I 3.56 (0.98, 1.40, 0.63, 0.55), II 2.61 (0.74, 0.96, 0.48, 0.43), III 2.16 (0.61, 0.67, 0.47, 0.41), IV 3.16 (0.86, 1.03, 0.73, 0.54). Opisthosoma oval, venter dark brown, dorsal scutum almost 1/2 the length of the opisthosoma. Spinnerets pale yellow.

Palp (Fig. 18A–C). Tibia almost as long as patella. Retrolateral tibial apophysis (RTA) as long as tibia, tip with three outgrowths. Cymbium almost as long as wide. Subtegulum (ST) unobvious. Tegulum oval. Conductor (C) helically twisted, straight, basal with an apophysis, triangle shaped, tip of conductor directed prolaterally at 9 o’clock position. Embolus (E) whip-like, start at ca 9 o’clock position, basal part of embolus with serration and an embolic apophysis (EA), embolus terminates at 8:30 o’clock position.

**Female paratype** (IZCAS-Ar44472) (Fig. 20D). Total length 2.36; carapace 1.12 long, 0.83 wide, opisthosoma 1.13 long, 0.77 wide. Eye sizes and interdistances: AME 0.03, ALE 0.04, PME 0.04, PLE 0.04, AME–AME 0.03, AME–ALE 0.04, PME–PME 0.02, PME–PLE 0.01, AME–PME 0.02, ALE–PLE 0.01. Chelicerae as in male. Leg measurements: I 2.76 (0.81, 1.06, 0.45, 0.44), II 2.20 (0.64, 0.83, 0.35, 0.38), III 1.97 (0.55, 0.62, 0.40, 0.40), IV 2.75 (0.71, 0.95, 0.63, 0.46). Opisthosoma oval, venter yellow-brown, dorsal scutum absent. Spinnerets pale yellow.

Epigyne (Fig. 19A, B). Epigynal plate ~ 2× longer than wide, fovea absent, with long, thin inflexible scape (SC), bearing small dorsal pit at the tip, length/width = 10/1. Copulatory openings (CO) almost oval, located in anterior part, separated by ~ 2× width. Copulatory ducts (CD) spaced by diameter anteriorly, adjoining near anterior margin of copulatory opening, and turning to tubular spermatheca (S) with ten loops. Fertilization ducts (FD) directed at 5 o’clock position from spermathecae.

##### Distribution.

Known only from the type locality.

##### Etymology.

The species is named after Li Jun, one of the 108 outlaws in the classical Chinese novel ‘Outlaws of the Marsh’; noun in apposition.

#### ﻿Genus Yuqilin gen. nov.

Taxon classificationAnimaliaAraneaeGnaphosidae

B8BD6535-8352-5306-B7F7-002FAF7B44C5

https://zoobank.org/A919C3D1-3351-4CBF-B749-4A19AE04BA78

##### Type species.

*Yuqilinlujunyi* sp. nov.

##### Diagnosis.

**Figure 21.**
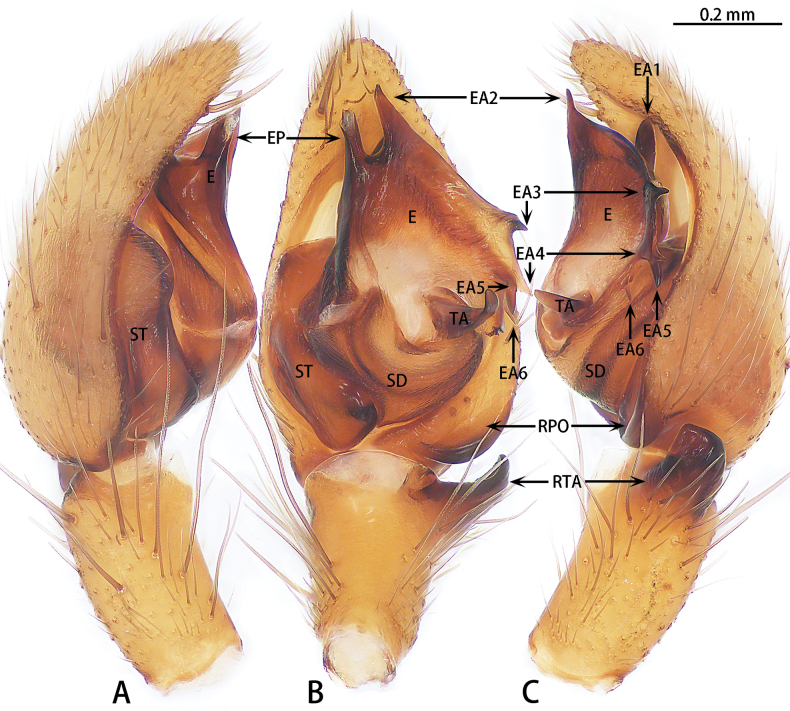
*Yuqilinlujunyi* sp. nov., holotype male **A** prolateral view **B** ventral view **C** retrolateral view. Abbreviations: E = embolus, EA1–6 = embolic apophysis 1–6, EP = embolus proper, RPO = retro-proximal cymbial outgrowth, RTA = retrolateral tibial apophysis, SD = sperm duct, ST = subtegulum, TA = tegular apophysis.

**Figure 22.**
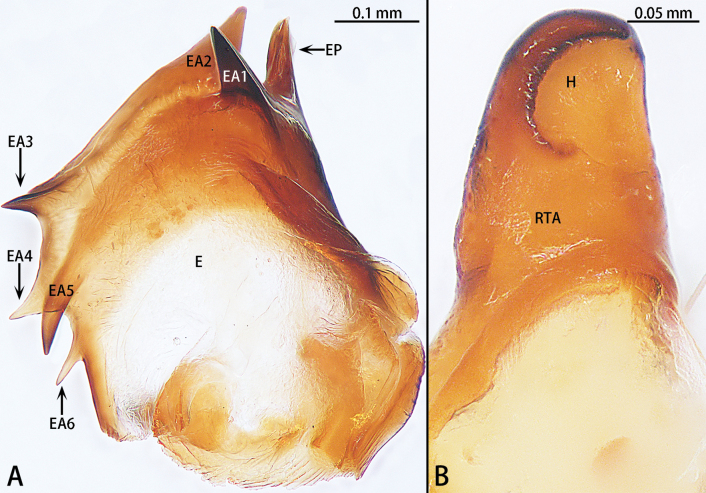
*Yuqilinlujunyi* sp. nov., paratype male **A** embolus, dorsal view **B** retrolateral tibial apophysis, dorsal view. Abbreviations: E = embolus, EA1–6 = embolic apophysis 1–6, EP = embolus proper, H = hood, RTA = retrolateral tibial apophysis.

**Figure 23.**
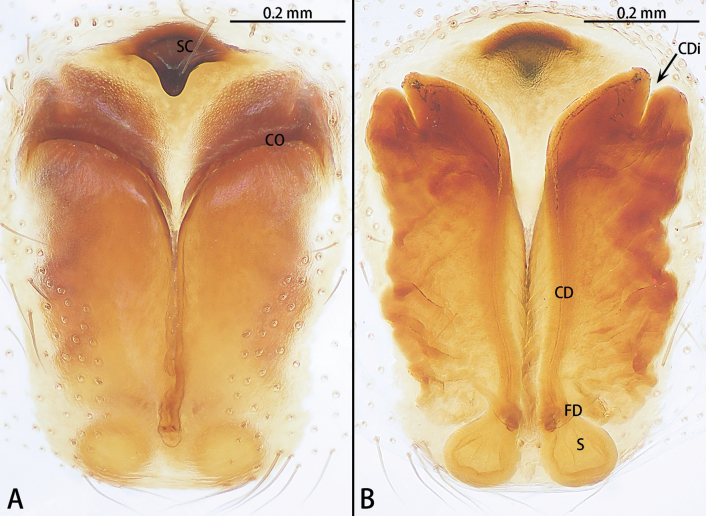
*Yuqilinlujunyi* sp. nov., paratype female **A** epigyne, ventral view **B** vulva, dorsal view. Abbreviations: CD = copulatory duct, CDi = copulatory duct anterior incision, CO = copulatory opening, FD = fertilization duct, S = spermatheca, SC = scape.

Females of *Yuqilin* gen. nov. resemble the *fallens* group in *Drassyllus* Chamberlin, 1922 (see Platnick and Shadab 1982: figs 9, 17) morphologically by the epigynal plate with a large scape anteriorly (Fig. 23A), wide copulatory ducts subparallel (Fig. 23B) and spermathecae subglobular, located posteriorly (Fig. 23B), but it differs in the following: epigynal plate without atrium (Fig. 23A) (vs present) and copulatory ducts with anterior incision (Fig. 23B) (vs absent). Males can be distinguished by the following characters: embolus as wide as bulb, wider than tegulum, with few apophyses, retro-proximal cymbial outgrowth present and condoctor absent (Figs 21B, 22A).

##### Description.

**Figure 24.**
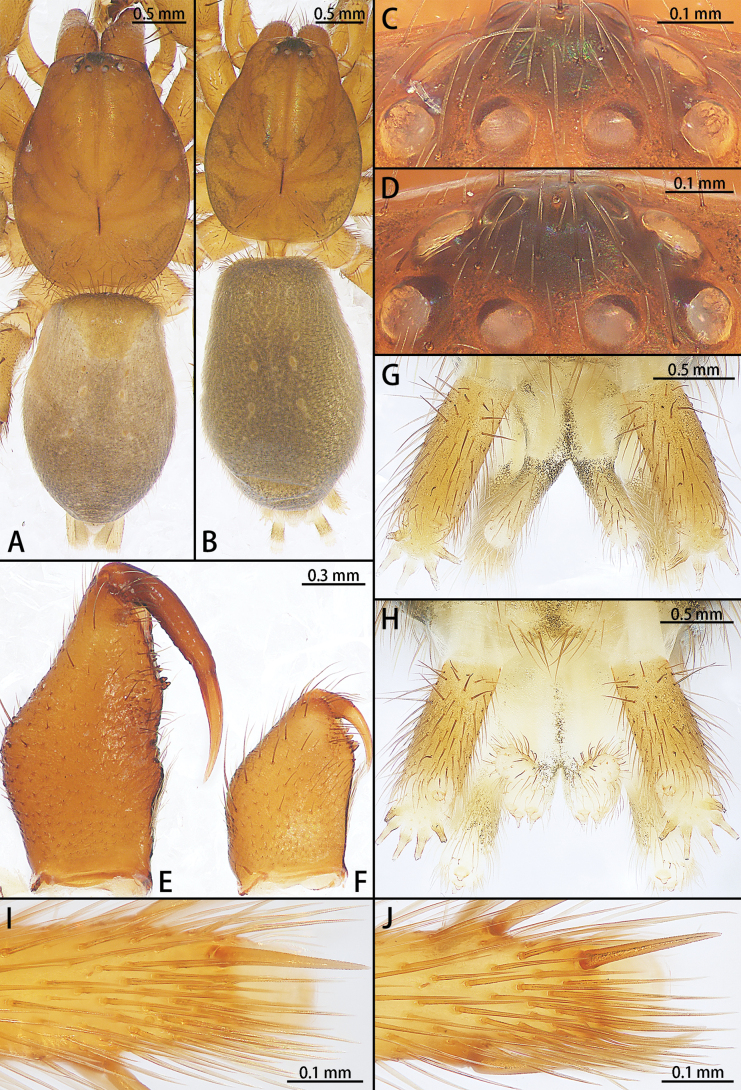
*Yuqilinlujunyi* sp. nov., male holotype (**A**, **C**, **E**, **G, I**) and female paratype (**B**, **D**, **F**, **H, J**) **A**, **B** habitus, dorsal view **C**, **D** eye area **E**, **F** chelicerae **G**, **H** spinnerets **I**, **J** metatarsi IV.

**Male** (Fig. 24A, C, E, G, I). Total length 3.96–5.21 (*n* = 6). Carapace red brown, with dark brown pattern, covered with few brown setae. Fovea longitudinal. Clypeus brown, covered with several setae. Chelicerae red-brown, with four promarginal and four retromarginal teeth. Endites pale brown. Labium pale brown, covered with brown setae. Sternum colored as endites, covered with brown setae. Legs brown, with a preening brush on metatarsi III and IV. Opisthosoma oval, venter brown with setae, dorsal scutum almost 1/3 the length of the opisthosoma. Spinnerets yellow brown.

Palp and palpal structures as in Figs 21A–C, 22A, B. Palpal femur almost 5× longer than patella, curved, tip with spines. Tibia with stout retrolateral tibial apophysis (RTA). Cymbium longer than wide, with retro-proximal cymbial outgrowing (RPO). Subtegulum (ST) obvious. Tegular apophysis (TA) present. Conductor (C) absent. Embolus (E) complex, almost triangle shaped, as wide as bulb. Embolus proper (EP) straight, directed anteriorly, with large retrolateral lamina with spine-like apophyses.

**Female** (Fig. 24B, D, F, H, J). Total length 4.01–5.51(*n* = 5). Habitus similar to those of male.

Epigynal plate (Fig. 23A, B) longer than wide, with scape (SC) located anteriorly. Copulatory openings (CO) located anteriorly, slit-like, slightly arched, almost touching each other. Copulatory duct (CD) wide, laminar, with anterior incision (CDi). Spermathecae (S) small, subglobular, located posteriorly.

##### Etymology.

The genus is named after Yuqilin, nickname for one of the 108 outlaws in the classical Chinese novel ‘Outlaws of the Marsh’; masculine in gender.

##### Composition.

The new genus currently includes only one species: *Yuqilinlujunyi* sp. nov.

##### Distribution.

China (Yunnan).

##### Comments.

This genus is not assigned to any of the known subfamilies.

#### ﻿ Yuqilin lujunyi sp. nov.

Taxon classificationAnimaliaAraneaeGnaphosidae

7414B720-47F2-517C-8122-3F79D5F9A6BE

https://zoobank.org/412EC3C4-0720-4249-BFD4-9AA591763A67

Figs 21A–C , 22A, B , 23A, B 24A–H

##### Type material.

***Holotype***: ♂ (IZCAS-Ar44487), China, Yunnan: Menglun Town: Xishuangbanna Nature Reserve, 21.9611°N, 101.1982°E, ca 790 m, 16–24.2006, Guo Zheng leg. ***Paratypes***: 5♂5♀ (IZCAS-Ar44488–Ar44497), same data as holotype.

##### Diagnosis.

Same as for the genus diagnosis.

##### Description.

**Male holotype** (Fig. 24A, C, E, G, I). Total length 4.33; carapace 2.18 long, 1.64 wide, opisthosoma 2.04 long, 1.36 wide. Eye sizes and interdistances: AME 0.07, ALE 0.10, PME 0.08, PLE 0.09, AME–AME 0.07, AME–ALE 0.02, PME–PME 0.08, PME–PLE 0.06, AME–PME 0.10, ALE–PLE 0.04. Anterior eye row recurved, posterior eye row straight. Chelicerae with four promarginal and four retromarginal teeth. Legs with long brown hair. Leg measurements: I 5.84 (1.66, 2.09, 1.22, 0.87), II 4.62 (1.34, 1.63, 0.93, 0.72), III 3.87 (1.11, 1.21, 0.93, 0.62), IV 6.47 (1.71, 2.22, 1.69, 0.85). Opisthosoma oval, venter brown with setae, dorsal scutum almost 1/3 the length of the opisthosoma. Spinnerets yellow brown.

Palp (Figs 21A–C, 22A, B). Femur almost 5× longer than patella, curved, tip with spines. Tibia almost 0.7 of patella length. Retrolateral tibial apophysis (RTA) almost 1.5× wider than long, tip with kind of hood (H). Cymbium almost 1.5× longer than wide, with retro-proximal cymbial outgrowing. Subtegulum (ST) large, almost 0.5 length of the cymbial length (prolateral view). Tegulum small. Tegular apophysis (TA) with wide base, ~ 2/3 of its length, tip claw-like. Conductor (C) absent. Embolus (E) complex, almost triangle shaped, as wide as bulb. Embolus proper (EP) straight, directed anteriorly, with large retrolateral lamina with six spine-like apophyses: one anterior (EA2), one dorsal (EA1), two retrolateral (EA3, 4) and two posteriors (EA5, 6).

**Female paratype** (IZCAS-Ar44488) (Fig. 24B, D, F, H, J). Total length 4.07; carapace 1.68 long, 1.28 wide, opisthosoma 2.48 long, 1.53 wide. Eye sizes and interdistances: AME 0.13, ALE 0.12, PME 0.12, PLE 0.11, AME–AME 0.02, AME–ALE 0.01, PME–PME 0.04, PME–PLE 0.02, AME–PME 0.06, ALE–PLE 0.02. Anterior eye row almost straight, posterior eye row recurved. Chelicerae with four promarginal and two retromarginal teeth. Leg measurements: I 4.27 (1.24, 1.54, 0.83, 0.66), II 3.55 (1.08, 1.21, 0.70, 0.56), III 3.32 (1.00, 1.21, 0.70, 0.56), IV 4.68 (1.23, 1.58, 1.21, 0.66). Opisthosoma oval, without dorsal scutum, venter yellow-brown with long brown setae. Spinnerets pale yellow.

Epigyne (Fig. 23A, B). Epigynal plate 1.5× longer than wide, with short scape (SC) located anteriorly. Copulatory openings (CO) located anteriorly, slit-like, slightly arched, each 0.5 of plate width. Copulatory duct (CD) wide, laminar (0.5 of plate width), with anterior incision (CDi). Spermathecae (S) small, subglobular, slightly wider than long, separated by ~ 1/5 of their width, located posteriorly. Fertilization ducts directed at 2 o’clock position from spermathecae.

##### Distribution.

Known only from the type locality.

##### Etymology.

The species is named after Lu Junyi, one of the 108 outlaws in the classical Chinese novel ‘Outlaws of the Marsh’; noun in apposition.

## ﻿Discussion

Adding the new species reported here, a total of 20 gnaphosid spider species are reported from XTBG. A checklist of XTBG gnaphosid spiders follows; for a complete list of taxonomic references see WSC (2023).

1. *Allomicythuskamurai* Ono, 2009
2. *Allomicythussuochao* sp. nov.
3. *Coillinayogeshi* (Gajbe, 1993)
4. *Hitobiacancellata* Yin, Peng, Gong & Kim, 1996
5. *Hitobiamenglong* Song, Zhu & Zhang, 2004
6. *Hitobiaunifascigera* (Bösenberg & Strand, 1906)
7. *Hitobiayunnan* Song, Zhu & Zhang, 2004
8. *Hongkongialiutang* sp. nov.
9. *Hongkongiareptrix* Deeleman-Reinhold, 2001
10. *Hongkongiawuae* Song & Zhu, 1998
11. *Laroniuserawan* Platnick & Deeleman-Reinhold, 2001
12. *Meizhelanmuhong* sp. nov.
13. *Sernokorbaruanxiaoer* sp. nov.
14. *Synaphosusevertsi* Ovtsharenko, Levy & Platnick, 1994
15. *Synaphosusfemininis* Deeleman-Reinhold, 2001
16. *Synaphosusleiheng* sp. nov.
17. *Synaphosuslijun* sp. nov.
18. *Yuqilinlujunyi* sp. nov.
19. *Zelotesshantae* Tikader, 1982
20. *Zelotesyani* Yin, Bao & Zhang, 1999

## Supplementary Material

XML Treatment for Allomicythus

XML Treatment for Allomicythus kamurai

XML Treatment for Allomicythus suochao

XML Treatment for Hongkongia

XML Treatment for Hongkongia liutang

XML Treatment for Hongkongia wuae

XML Treatment for Meizhelan

XML Treatment for Meizhelan muhong

XML Treatment for Sernokorba

XML Treatment for Sernokorba ruanxiaoer

XML Treatment for Synaphosus

XML Treatment for Synaphosus leiheng

XML Treatment for Synaphosus lijun

XML Treatment for Yuqilin

XML Treatment for Yuqilin lujunyi

## ﻿Additional information

### Author ORCIDs

Yejie Lin https://orcid.org/0000-0002-6789-2731

Shuqiang Li https://orcid.org/0000-0002-3290-5416

### Data availability

All of the data that support the findings of this study are available in the main text.
